# An Engineered HR1‐Stem Helix‐HR2 Trimeric Platform for Developing Broad‐Spectrum Coronavirus Vaccines

**DOI:** 10.1002/advs.77384

**Published:** 2026-09-01

**Authors:** Xikui Sun, Junhao Fan, Xiaolu Xie, Yuting Shi, Xiaoqing Liu, Tiefeng Xu, Sidi Yang, Keda Shi, Chengshu Xie, Shixiong Li, Siwen Chen, Ruoxi Cai, Ping He, Yuzhen Ye, Tao Chen, Xiancai Ma, Chunmei Li, Jingyou Yu, Deyin Guo

**Affiliations:** ^1^ State Key Laboratory of Respiratory Disease National Clinical Research Center for Respiratory Disease Guangzhou Institute of Respiratory Health The First Affiliated Hospital of Guangzhou Medical University Guangzhou China; ^2^ Guangzhou National Laboratory Guangzhou International Bio‐Island Guangzhou China; ^3^ Key Laboratory of Tropical Disease Control of Ministry of Education Institute of Human Virology Department of Pathogen Biology and Biosecurity Zhongshan School of Medicine Sun Yat‐Sen University Guangzhou China; ^4^ College of Life Sciences Nankai University Tianjin China; ^5^ Centre For Infection and Immunity Study (CIIS) School of Medicine Shenzhen Campus of Sun Yat‐Sen University Shenzhen China

**Keywords:** broad‐spectrum vaccines, coronaviruses, stem helix

## Abstract

The rapid spread of immune‐evasive viral variants, exemplified by SARS‐CoV‐2, highlights the urgent need for broad‐spectrum coronavirus vaccines. Although multimeric display of the receptor‐binding domain (RBD) using exogenous scaffolds improve immune responses, such approaches may elicit off‐target immune responses against the scaffolds themselves and remain limited by rapid RBD antigenic drift. Here, we report the rational design of a self‐assembling trimeric subunit vaccine, termed RBD‐heptad repeat 1 (HR1)‐stem helix (SH)‐heptad repeat 2 (HR2) (RHS), which integrates the JN.1 RBD with a highly conserved SH epitope from SARS‐CoV‐2 within a native HR1‐HR2 trimeric scaffold via optimized linkers. RHS exhibits high structural stability, efficient trimerization, and enhanced antigen presentation. In mice, RHS elicits robust humoral and cellular immune responses, including potent cross‐ neutralizing antibodies against diverse SARS‐CoV‐2 variants and pan‐betacoronavirus SH‐specific antibodies. In K18‐hACE2 mice, RHS confers strong protection against both antigen‐matched and antigen‐mismatched Omicron variants, while intranasal immunization induces potent mucosal IgA and IgG responses. Furthermore, the modular RHS platform is readily adaptable to antigens from SARS‐CoV and MERS‐CoV, underscoring its versatility for pan‐coronavirus vaccine development. Together, these findings establish RHS as a generalizable strategy for overcoming viral immune evasion and advancing next‐generation vaccine design.

## Graphical abstract

Sun et al. report a self‐assembling trimeric subunit vaccine platform termed RHS, which integrates the JN.1 receptor‐binding domain (RBD) with the heptad repeat 1 (HR1), stem helix (SH), and heptad repeat 2 (HR2) regions. The SARS‐CoV‐2 RHS vaccine elicits variant‐reactive RBD‐specific antibodies and cross‐reactive SH‐specific antibodies against betacoronaviruses. The platform also supports adaptation to SARS‐CoV, MERS‐CoV, and ovalbumin (OVA) antigens, demonstrating its modularity and potential for pan‐coronavirus vaccine development.

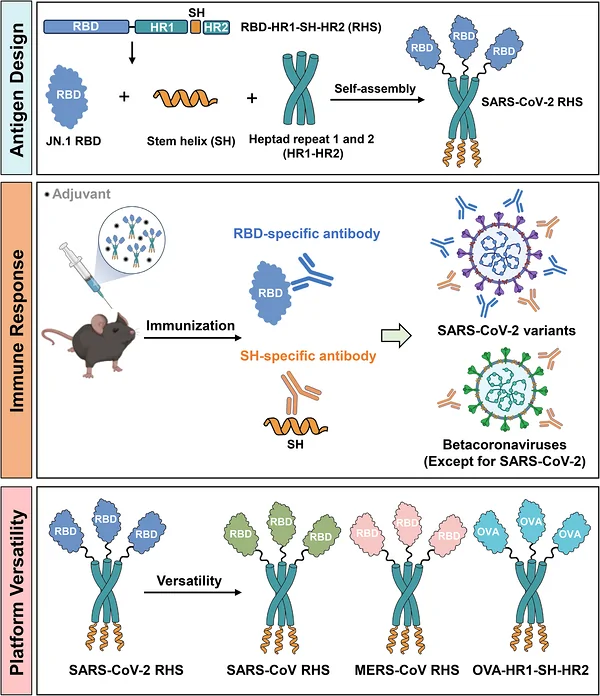

## 1 Introduction

Over the past two decades, the emergence of three highly pathogenic coronaviruses, severe acute respiratory syndrome coronavirus (SARS‐CoV), Middle East respiratory syndrome coronavirus (MERS‐CoV), and severe acute respiratory syndrome coronavirus 2 (SARS‐CoV‐2), has underscored the urgent need for broadly protective coronavirus vaccines [1]. This need is intensified by the rapid and continual evolution of SARS‐CoV‐2, as successive variants erode the effectiveness of existing vaccines. The recent dominance of the JN.1 lineage and its immune‐evasive sublineages (e.g., KP.2, KP.3, KP.2.3, XEC, KP.3.1.1, XFG, and NB.1.8.1) exhibit markedly enhanced immune escape [2, 3, 4], reduced serum neutralization, and increasing global prevalence, posing substantial challenges for current immunization strategies. Moreover, immune imprinting from vaccines based on ancestral or XBB‐lineage strains further constrains the breadth of antibody responses against these highly mutated JN.1‐sublineages, highlighting the critical limitations of variant‐chasing strategies [5, 6]. Consequently, developing universal vaccine platforms that can elicit broad and durable protection against diverse coronaviruses has become a paramount scientific objective.

Subunit vaccines targeting the receptor‐binding domain (RBD) of coronaviral spike (S) can induce potent neutralizing antibodies (NAbs), but often require engineered multimerization to achieve optimal immunogenicity. Current multimerization strategies employing exogenous scaffolds, including human Fc fragments, T4 fibritin foldon, the coiled‐coil domain of General Control Nonderepressible 4, or various nanoparticles, effectively mimic the native viral structure and enhance B‐cell receptor (BCR) crosslinking [7, 8, 9, 10]. However, these exogenous elements may be immunogenic, potentially diverting immune responses and raising safety concerns for clinical translation [11]. An alternative approach is to exploit the endogenous structural machinery of viruses themselves. The S2 element mediates membrane fusion during coronavirus entry, in which the highly conserved heptad repeat 1 (HR1) and heptad repeat 2 (HR2) regions naturally self‐assemble into a stable six‐helix bundle (6‐HB) through hydrophobic interactions [12, 13], providing an ideal endogenous scaffold for rational antigen design.

The RBD continues to accumulate mutations that enable escape from many previously identified NAbs [14]. In contrast, the S2 region is more conserved. NAbs targeting the fusion peptide (FP), HR1‐HR2 region, or the stem helix (SH) display cross‐reactive neutralization across diverse betacoronaviruses (β‐coronaviruses) [15, 16, 17, 18]. The SH, located immediately upstream of HR2 and critical for forming 6‐HB, represents a particularly attractive target [15]. Multiple broadly NAbs including S2P6, CV3‐25, CC99.103, and CC40.8 recognize this highly conserved epitope and neutralize a wide spectrum of β‐coronaviruses [18, 19, 20, 21], underscoring its potential for pan‐coronavirus vaccine development. Incorporating this conserved epitope into a rationally engineered antigen may therefore be able to redirect immunity away from variable surfaces and toward a universal target.

In this study, we developed a self‐assembling trimeric fusion protein vaccine termed RBD‐HR1‐SH‐HR2 (RHS). By integrating a conserved SH epitope within a native HR1‐HR2 trimer scaffold, the RHS design simultaneously enhances antigen stability, multimerization, and both the breadth and magnitude of antibody responses. Importantly, this modular strategy is readily adaptable to antigens from SARS‐CoV and MERS‐CoV, underscoring the versatility and translational potential of the RHS platform for universal coronavirus vaccine development.

## 2 Results

### 2.1 SARS‐CoV‐2 RHS Self‐Assembles Into a Trimeric Protein

**FIGURE 1:**
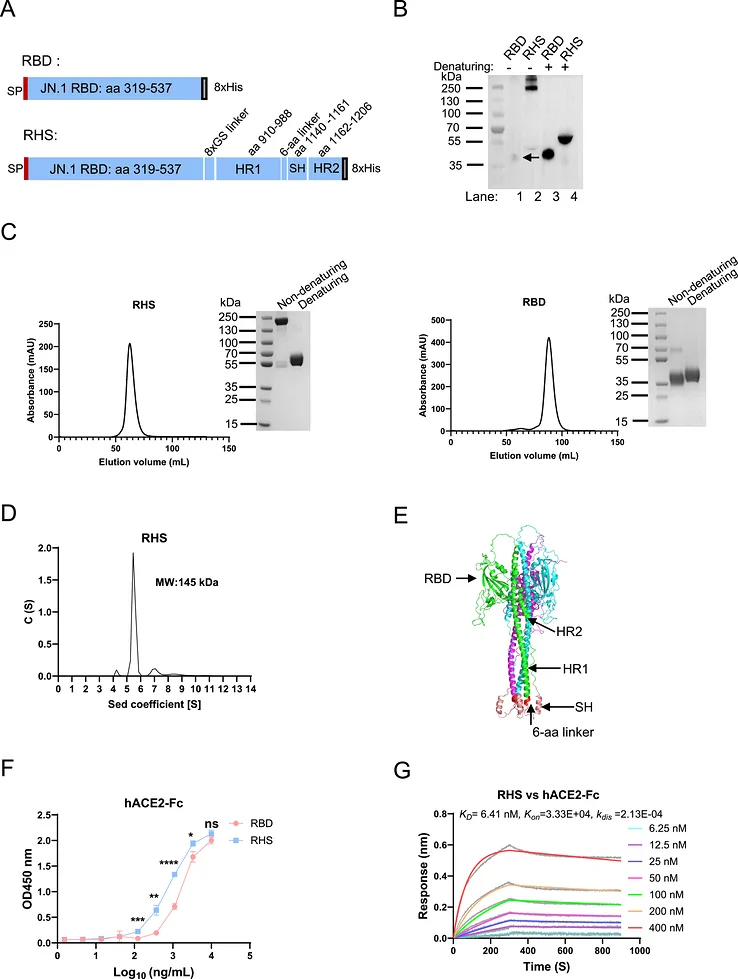
Design and characterization of RHS trimer protein. (A) The schematic diagram of JN.1 RBD and RHS design. The RHS fusion protein was constructed by fusing signal peptide (SP), JN.1 RBD (aa 319‐537), 8×GS linker, HR1 (aa 910‐988), 6‐aa linker (SGGRGG), SH (aa 1140‐1161), HR2 (aa 1162‐1206), and an 8× His tag. (B) Western blot analysis of JN.1 RBD and RHS expression in culture supernatants of HEK293T cells 48 h post‐transfection under denaturing and non‐denaturing conditions. For denaturing conditions, supernatants were mixed with 5 × SDS‐PAGE protein loading buffer containing β‐mercaptoethanol and heated at 100°C for 10 min prior to SDS‐PAGE analysis. For non‐denaturing conditions, supernatants were mixed with 5 × SDS‐PAGE protein loading buffer without β‐mercaptoethanol and analyzed without heat treatment. The weak JN.1 RBD signal under non‐denaturing conditions is indicated by a black arrow. The weak JN.1 RBD signal under non‐denaturing conditions is likely due to burial of the His tag within the folded RBD. (C) SEC profiles of RHS (left) and JN.1 RBD (right) using a HiLoad 16/600 Superdex 200 pg column under native, non‐denaturing conditions. The UV 280 nm absorbance curves are indicated. The SDS‐PAGE analysis of the purified RHS (left) and JN.1 RBD (right) proteins in non‐denaturing and denaturing conditions were shown on the right of each corresponding SEC. (D) Ultracentrifugation sedimentation profiles of RHS protein. (E) The structure of RHS predicted by AlphaFold3. (F) The binding activity of JN.1 RBD and RHS to hACE2‐Fc at the indicated concentrations determined by ELISA. Data points represent mean ± standard deviation (SD) (*n* = 3) from three independent experiments. (G) Binding kinetics of human angiotensin‐converting enzyme 2 (hACE2)‐Fc to RHS were analyzed by BLI. The values of association rate constant (*K*_on_), dissociation rate constant (*K*_dis_), and equilibrium dissociation constant (*K*_D_) are shown. Western blot, SEC, SDS‐PAGE, AUC, and BLI data were repeated in at least two independent experiments with similar results. The data are shown from one representative experiment. Statistical comparisons were performed by unpaired one‐tailed Student's *t*‐test (F). *p < 0.05; **p < 0.01; ***p < 0.001; ****p < 0.0001; ns, no significant difference.

Multivalent antigen presentation consistently demonstrate enhanced immunogenicity compared with their monomeric counterparts [22]. In addition, incorporating antigenic epitopes targeted by broadly NAbs can enhance the breadth of vaccine‐elicited immunity [23]. Given the recent dominance of SARS‐CoV‐2 Omicron JN.1 lineage and its immune‐evasive sublineages, updating antigen design is urgently required. To promote RBD trimerization while incorporating the broadly neutralizing SH epitope, we engineered a JN.1 S protein based fusion protein termed RHS, which consists of JN.1 RBD, an 8 × GS linker, HR1, a 6‐amino‐acid (aa) linker, SH, HR2, and a C‐terminal 8 × His tag (Figure 1A). The full length of RHS protein contains 396 aa with a predicted molecular mass of 43.8 kDa. To assess whether RHS forms a trimer, HEK293T cells were transfected with expression plasmids encoding JN.1 RBD or RHS, and the culture supernatants were collected for Western blot analysis (Figure 1B). Under both non‐denaturing and denaturing conditions, the molecular weight of JN.1 RBD remained almost unchanged. In contrast, RHS appeared predominantly as polymers (∼200 kDa) under non‐denaturing conditions, but resolved into a monomeric form (∼50 kDa) upon heat treatment and reduction, consistent with trimer formation under native conditions. Furthermore, RHS was expressed in Expi293F cells and purified by TALON affinity chromatography followed by size‐exclusion chromatography (SEC), which yielded a single peak corresponding to a multimeric protein (Figure 1C, left). Sodium dodecyl sulfate‐polyacrylamide gel electrophoresis (SDS‐PAGE) analysis also indicated RHS self‐assembled into a multimeric protein (Figure 1C, left). In contrast, the SEC profile of JN.1 RBD displayed one major peak and a minor peak (Figure 1C, right). SDS‐PAGE analysis revealed the majority of RBD existed as monomer, while a small fraction forming dimers in non‐denaturing conditions. Analytical ultracentrifugation (AUC) showed that RHS has an apparent molecular mass of ∼145 kDa, approximately three times that of a monomer, confirming that RHS was a trimer protein (Figure 1D). AlphaFold 3 structural prediction of RHS suggested that the HR1‐SH‐HR2 domain formed a 6‐HB scaffold with RBD connected through a flexible linker (Figure 1E). Enzyme‐linked immunosorbent assay (ELISA) suggested that RHS bound human angiotensin‐converting enzyme 2 (hACE2)‐Fc with higher affinity than monomeric RBD (Figure 1F). We also examined the binding kinetics of hACE2‐Fc to RBD and RHS using bio‐layer interferometry (BLI) (Figure 1G; Figure S1). The result indicated that RHS had higher binding activity to hACE2‐Fc than monomeric RBD. These measurements establish that RHS forms a stable trimeric structure while retaining an ACE2‐accessible RBD conformation.

### 2.2 RHS Vaccine Induces More Broad‐Spectrum Antibody Responses and More Potent CD8^+^ T‐Cell Immune Responses Than RBD

To evaluate the immunogenicity of RHS antigen, 6–8‐week‐old female BALB/c mice received three intramuscular (IM) immunizations of RHS protein at doses of 0.2, 1, or 10 µg formulated with AddaVax adjuvant (Figure 2A). The JN.1 RBD served as a control immunogen. JN.1 RBD‐specific IgG titers were determined 2 weeks after each immunization. At low doses (0.2 µg), RHS elicited significantly higher IgG titers than RBD after each immunization (Figure 2B). At the medium (1 µg) and high (10 µg) doses, RHS also induced more robust antibody responses than RBD after prime and first boost immunization. Following the second boost immunization of medium and high dosages, the IgG titers all reached plateau so that there was no significant difference. To assess sequence variation among circulating SARS‐CoV‐2 variants, RBD sequences from SARS‐CoV‐2 wild‐type (WT), SARS‐CoV‐2 D614G variant, and SARS‐CoV‐2 Omicron subvariants BA.5, XBB.1.5, EG.5.1, BA.2.86, JN.1, SLip, KP.2, KP.3, KP.2.3, LB.1, KP.3.1.1, XEC, XFG, and NB.1.8.1 were aligned (Figure 2C). Multiple SARS‐CoV‐2 Omicron BA.2.86/JN.1 derived subvariants have emerged and accumulated additional mutations, particularly in recently circulating subvariants such as XFG and NB.1.8.1. To assess the neutralization breadth of the JN.1 RHS‐based vaccine, pseudovirus neutralization assays were performed after the second boost to determine half‐maximal neutralizing titers (NT_50_) (Figure 2D). At the 0.2 µg dose, RBD failed to elicit detectable NAbs against SARS‐CoV‐2 D614G variant, and SARS‐CoV‐2 Omicron subvariants BA.5, XBB.1.5, EG.5.1, XFG, and NB.1.8.1. It induced only low titers (< 500) against BA.2.86/JN.1‐related Omicron subvariants, including SLip, KP.2, KP.3, KP.2.3, LB.1, KP.3.1.1, and XEC. In contrast, RHS at 0.2 µg elicited detectable NAbs against all tested variants, with titers exceeding 1750 against BA.2.86/JN.1‐related Omicron subvariants, and detectable neutralization against XFG (NT_50_ = 357) and NB.1.8.1 (NT_50_ = 3306). At 1 and 10 µg doses, RHS consistently induced stronger and broader neutralizing responses than RBD across all variants tested, including SARS‐CoV‐2 D614G variant, and SARS‐CoV‐2 Omicron XFG, and NB.1.8.1 variants. Radar plot analysis confirmed that RHS elicited substantially broader antibody responses than monomer RBD, especially at low and medium doses (Figure 2E). To further validate these findings, authentic virus neutralization assays were performed using Omicron JN.1 and EG.5.1 variants. Serum neutralizing activity was measured by focus reduction neutralization test (FRNT). Consistent with pseudovirus assays, RHS elicited higher NTs against JN.1 than RBD at all tested doses (Figure 2F). Notably, RBD induced minimal or undetectable neutralizing activity against EG.5.1, whereas RHS induced detectable neutralization at a dose of 0.2 µg and potent responses at 1 and 10 µg, confirming its superior breadth and potency against the tested authentic variants.

FIGURE 2The immunogenicity of JN.1 RHS in BALB/c mice. (A) Immunization schedule. A 6–8‐week‐old female BALB/c mice were immunized intramuscularly with three doses of JN.1 RBD or RHS (0.2, 1, or 10 µg) formulated with AddaVax adjuvant. An equal volume of PBS was administered to control mice. The blood samples were collected 2 weeks after each immunization for determining the antibody response. At Week 8, the mice were sacrificed and spleens were collected for cellular immunity assessment. (B) JN.1 RBD‐specific IgG antibody response measured by ELISA 2 weeks after each immunization. Median ± interquartile range (IQR) are shown (*n* = 5). (C) Alignment of RBD amino acid sequences from SARS‐CoV‐2 wild‐type strain (WT), SARS‐CoV‐2 D614G variant, and SARS‐CoV‐2 Omicron subvariants BA.5, XBB.1.5, EG.5.1, BA.2.86, JN.1, SLip, KP.2, KP.3, KP.2.3, LB.1, KP.3.1.1, XEC, XFG, and NB.1.8.1. Amino acid substitutions relative to the WT sequence are highlighted in blue. (D) Half‐maximal neutralizing titers (NT_50_) determined by SARS‐CoV‐2 D614G variant and SARS‐CoV‐2 Omicron subvariants BA.5, XBB.1.5, EG.5.1, BA.2.86, JN.1, SLip, KP.2, KP.3, KP.2.3, LB.1, XEC, KP.3.1.1, XFG, and NB.1.8.1 pseudotyped viruses after last immunization. Median ± IQR are shown (*n* = 5). (E) Radar plot showing the spectrum of neutralization activity of JN.1 RBD and RHS at different doses. Data are presented as mean ± SD (*N* = 5). (F) Half‐maximal focus reduction neutralization titers (FRNT_50_) against authentic SARS‐CoV‐2 Omicron JN.1 and SARS‐CoV‐2 Omicron EG.5.1 variants. Median ± IQR are shown (*n* = 5). (G,H) The immune response of JN.1 S peptide‐specific CD4^+^ T cell (G) and CD8^+^ T (H) cell responses in splenocytes were detected by intracellular cytokine staining (ICS). Median ± IQR are shown (*n* = 4 for the PBS group, *n* = 5 for all other groups). Animal experiments were repeated at least twice independently with similar results. ELISA and pseudovirus neutralization, and authentic virus neutralization assays were performed using duplicate technical wells for each serum sample. Statistical comparisons were performed by one‐way ANOVA with Tukey's multiple‐comparison test (B, F–H). *p < 0.05; **p < 0.01; ***p < 0.001; ****p < 0.0001; ns, no significant difference.

**Figure:**
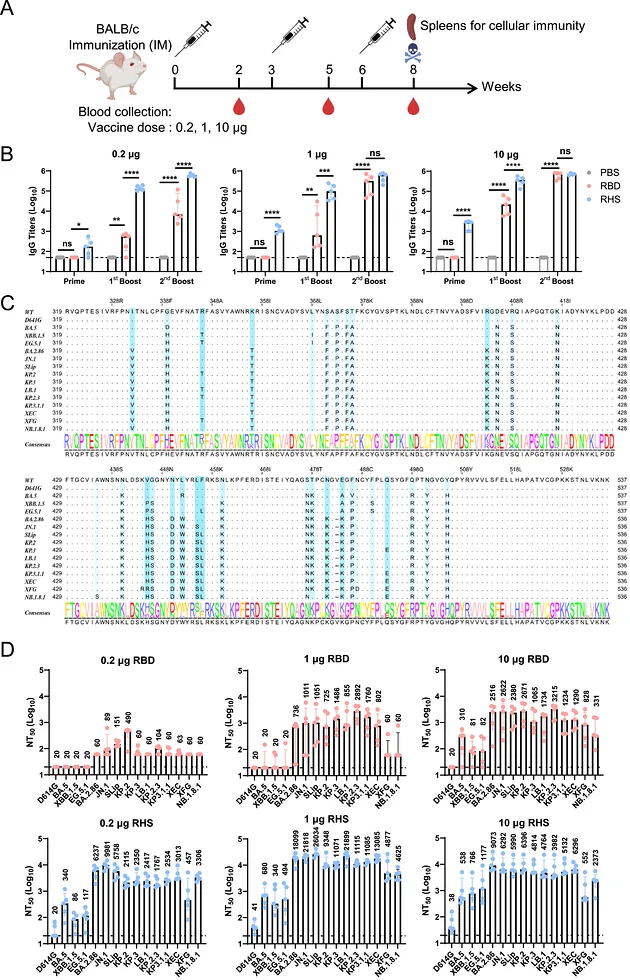

**Figure:**
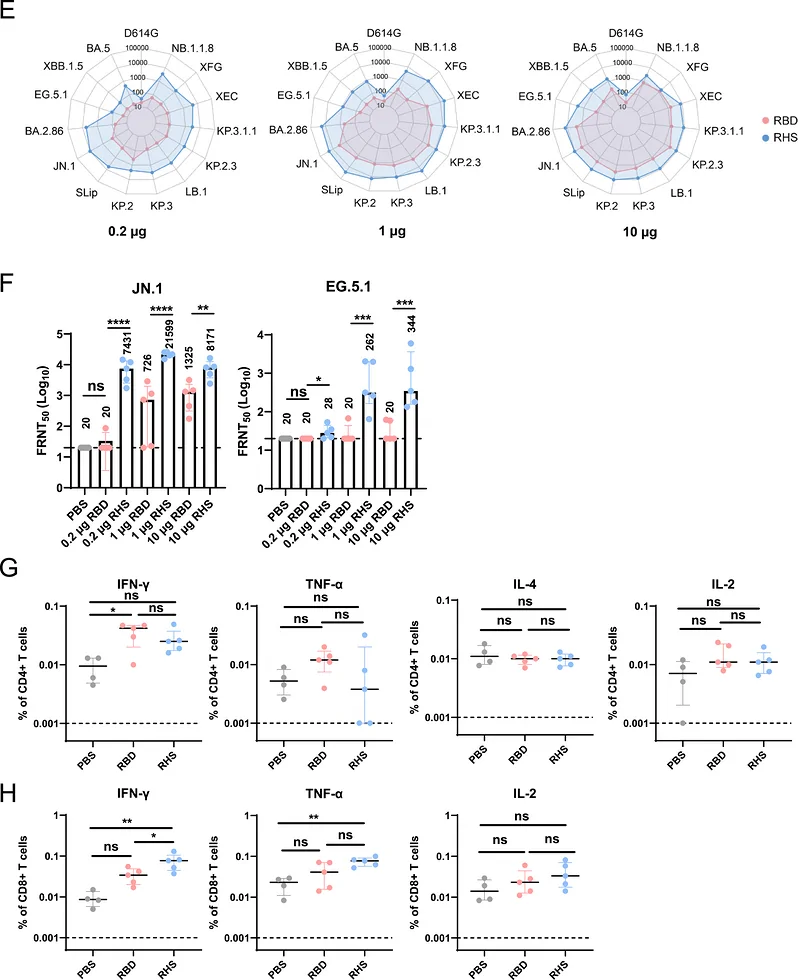

To characterize the cellular immune response, splenocytes were collected two weeks after the final immunization and subjected to interleukin‐4 (IL‐4) and interferon‐γ (IFN‐γ) enzyme‐linked immunospot (ELISpot) assays. No significant difference was observed for JN.1 RBD and RHS groups (Figure S2A). To further evaluate CD4^+^ and CD8^+^ T cell‐mediated immune responses, intracellular cytokine staining (ICS) assays were performed. The results indicated no statistically significant differences in the induction of IFN‐γ, tumor necrosis factor‐α (TNF‐α), interleukin‐2 (IL‐2), or IL‐4 in CD4^+^ T cells (Figure 2G; Figure S2B). However, RHS elicited significantly stronger IFN‐γ response in CD8^+^ T cells than phosphate‐buffered saline (PBS) and RBD groups. RHS also elicited significantly stronger TNF‐α response than PBS group, although the difference relative to the RBD group was not statistically significant. No significant differences in IL‐2 production were detected in CD8^+^ T cells (Figure 2H). Together, these data indicate that RHS induces robust CD8^+^ T cell‐mediated immunity in addition to a strong humoral response.

### 2.3 SH Epitope Enhances Both Breadth and Magnitude of the Antibody Response

The SH (aa 1140‐1161) epitope is highly conserved across β‐coronavirus (Figure 3A, left panel). The emerging SARS‐CoV‐2 Omicron BA.2.86/JN.1 lineage and its sublineages harbor a P1143L substitution in SH (Figure 3A, right panel). SH‐targeting antibodies CV3‐25, S2P6, and CC99.103 showed broad neutralization against β‐coronaviruses [18, 19, 21]. The peptide ELISA confirmed that the P1143L mutation in BA.2.86/JN.1 variants did not impair the binding of SH‐targeting antibodies (Figure 3B). CV3‐25 exhibited broad neutralizing activity against sarbecoviruses, including emerging BA.2.86/JN.1‐derived Omicron subvariants such as SLip, KP.2, KP.3, KP.2.3, LB.1, KP.3.1.1, and XEC (Figure S3). The SH‐specific NAbs prevent infection mainly through inhibition of membrane fusion [18]. CV3‐25 efficiently inhibited membrane fusion across all tested SARS‐CoV‐2 variants, whereas the RBD‐targeting antibody CB6, which overlaps with the hACE2 binding site, inhibited only the D614G variant (Figure S4), arguing for the potential of SH as an epitope for pan‐sarbecovirus vaccine development. Therefore, SH epitope was incorporated into RHS antigen, which was within a native HR1‐HR2 trimeric scaffold (Figure 1E). To determine whether SH or 6‐aa linker participated in the assembly of RHS trimer, we constructed two deletion mutants: RHSΔ6‐aa (6‐aa linker deletion) and RHSΔSH (SH deletion), which were expressed and validated by Western blot, SEC, SDS‐PAGE, AUC, AlphaFold 3 modeling, and ELISA (Figure 3C,D; Figure S5). The results demonstrated that neither 6‐aa linker nor SH contributed to RHS trimer and the binding to hACE2‐Fc. To determine whether the SH epitope in RHS could be accessible by SH‐specific antibodies, we used ELISA assay to detect their binding. The results showed that antibodies CV3‐25, S2P6, and CC99.103 had a strong binding affinity to RHS, which was almost completely diminished in RBD and RHSΔSH groups (Figure 3E). To determine whether SH epitope enhanced the immunogenicity of RHS, 6–8‐week‐old female C57BL/6 mice were immunized with three doses of 10 µg JN.1 RBD, RHS, or RHSΔSH formulated with AddaVax adjuvant (Figure 3F). The ELISA assay showed that RHS and RHSΔSH could induce significantly higher RBD‐specific antibody titers than RBD after each of immunization (Figure 3G), whereas the difference was not significant between RHS and RHSΔSH. NT_50_ values were assessed after the first booster immunization. JN.1 RBD induced undetectable neutralizing activity, whereas both RHS and RHSΔSH induced robust responses with titers exceeding 1000 (Figure 3H). No significant difference was observed between RHS and RHSΔSH. Following the second booster immunization, RHS elicited substantially stronger neutralizing antibody responses than RBD against SARS‐CoV‐2 Omicron subvariants JN.1, KP.2.3, XEC, KP.3.1.1, XFG, NB.1.8.1, EG.5.1, XBB.1.5, BA.5, and SARS‐CoV‐2 D614G variant (Figure 3I). In addition, RHS outperformed RHSΔSH against most tested Omicron variants. To determine whether the SH epitope could elicit a broad antibody response, peptide ELISA revealed that RHS induced cross‐reactive antibodies recognizing the SH of SARS‐CoV‐2 Omicron JN.1 variant, SARS‐CoV, MHV, OC43, HKU1, and MERS‐CoV, while no specific binding was observed for JN.1 RBD and RHSΔSH (Figure 3J). Moreover, only sera from RHS‐immunized mice efficiently inhibited CV3‐25 binding to the JN.1 S protein, suggesting RHS elicited antibodies that share epitopes with CV3‐25 and likely contributed to the breadth of NAbs (Figure 3K). Consistently, only RHS elicited detectable neutralization against SARS‐CoV, while no neutralization against MERS‐CoV was observed in any group (Figure 3L). Together, these results demonstrate that the SH epitope enhances both breadth and magnitude of the immune responses.

FIGURE 3SH epitope enhancing the immune effect of RHS. (A) Alignment of SH sequence from β‐coronavirus, including SARS‐CoV‐2 variants, SARS‐CoV, MERS‐CoV, human coronavirus OC43 (OC43), human coronavirus HKU1 (HKU1), mouse hepatitis virus (MHV), and SARS‐related coronavirus. (B) The peptide ELISA assay determining the binding of antibodies CV3‐25, CC99.103, and S2P6 to the representative beta‐coronavirus SH peptides. Data points represent mean ± SD (*n* = 3) from three independent experiments. The half‐maximal effective concentration (EC_50_) values were calculated using GraphPad Prism version 10. (C) The schematic diagram of RHSΔ6‐aa and RHSΔSH mutants. (D) SDS‐PAGE analysis of purified RHS, RHSΔ6‐aa, and RHSΔSH protein in non‐denaturing and denaturing conditions. For denaturing conditions, samples were mixed with 5 × SDS‐PAGE protein loading buffer containing β‐mercaptoethanol and heated at 100°C for 10 min prior to SDS‐PAGE analysis. For non‐denaturing conditions, samples were mixed with 5 × SDS‐PAGE protein loading buffer without β‐mercaptoethanol and analyzed without heat treatment. The experiments were repeated twice independently with similar results, and data from one representative experiment are shown. (E) Binding of antibodies CV3‐25, CC99.103, and S2P6 to RBD, RHS, and RHSΔSH protein at indicated concentration determined by ELISA from three independent experiments. Data points represent mean ± SD (*n* = 3). (F) Immunization schedule. A 6–8‐week‐old female C57BL/6 mice were immunized intramuscularly with three doses of 10 µg JN.1 RBD, RHS, or RHSΔSH formulated with AddaVax adjuvant. An equal volume of PBS was administered to control mice. The serum samples were collected 2 weeks after each immunization for determining the antibody response. (G) JN.1 RBD‐specific IgG antibody measured by ELISA after each immunization. Median ± IQR are shown (*n* = 5). (H) NT_50_ were determined by Omicron JN.1 variants pseudotyped viruses after first boost immunization. Median ± IQR are shown (*n* = 5). I) NT_50_ after second boost immunization were determined by D614G variant and Omicron subvariants JN.1, KP.2.3, XEC, KP.3.1.1, XFG, NB.1.8.1, BA.5, XBB.1.5, and EG.5.1 pseudotyped viruses. Median ± IQR are shown (*n* = 5). J) SARS‐CoV, JN.1, OC43, HKU1, MHV, and MERS‐CoV SH‐specific antibody titers determined by peptide ELISA after second boost immunization. Median ± IQR are shown from two experiments (*n* = 10). (K) The competitive ELISA analysis of the sera from JN.1 RBD, RHS, and RHSΔSH inhibiting CV3‐25 binding to JN.1 S protein after first immunization. Median ± IQR are shown (*n* = 5). L) NT_50_ against SARS‐CoV and MERS‐CoV pseudoviruses after the second boost. Data are presented as mean ± SD (*n* = 5). Animal experiments were repeated at least twice independently with similar results, and data from one representative experiment are shown. ELISA and pseudovirus neutralization assays were performed using duplicate technical wells for each serum sample. Statistical comparisons were performed by one‐way ANOVA with Tukey's multiple‐comparison test (B, G–K) or two‐way ANOVA (E). *p < 0.05; **p < 0.01; ***p < 0.001; ****p < 0.0001; ns, no significant difference.

**Figure:**
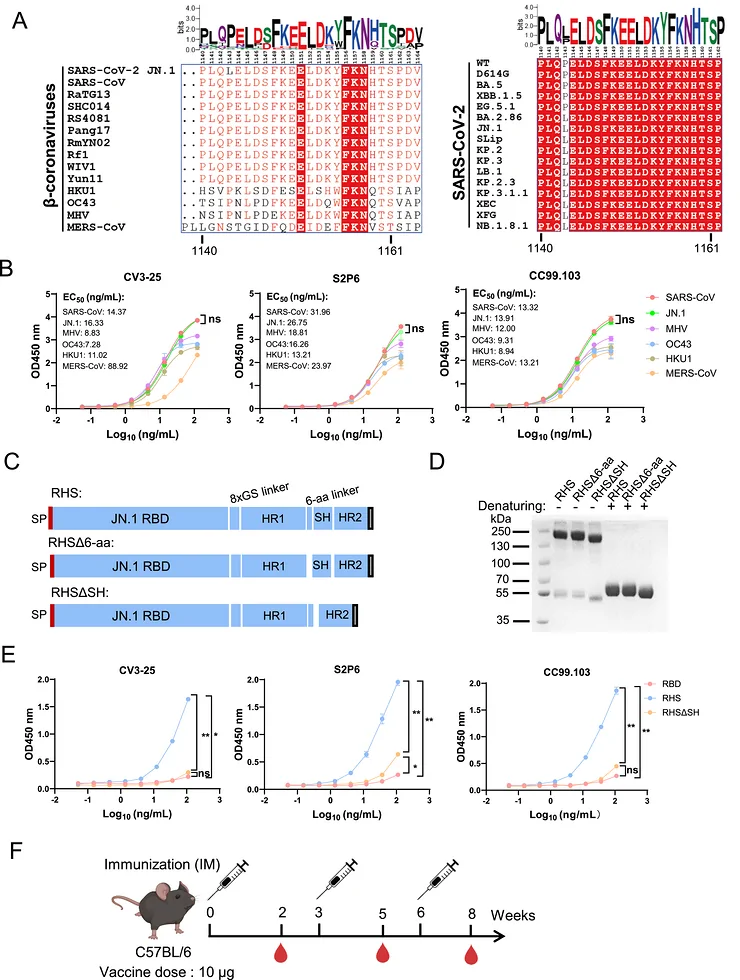

**Figure:**
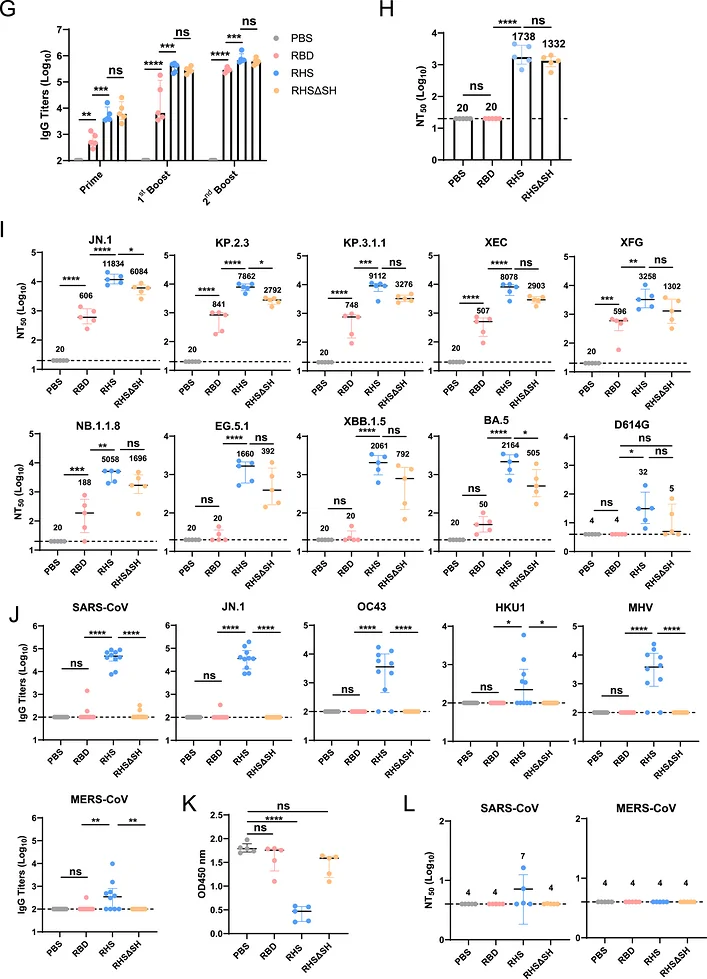

### 2.4 The Linker Between RBD and HR in RHS Influences Immunogenicity

**FIGURE 4:**
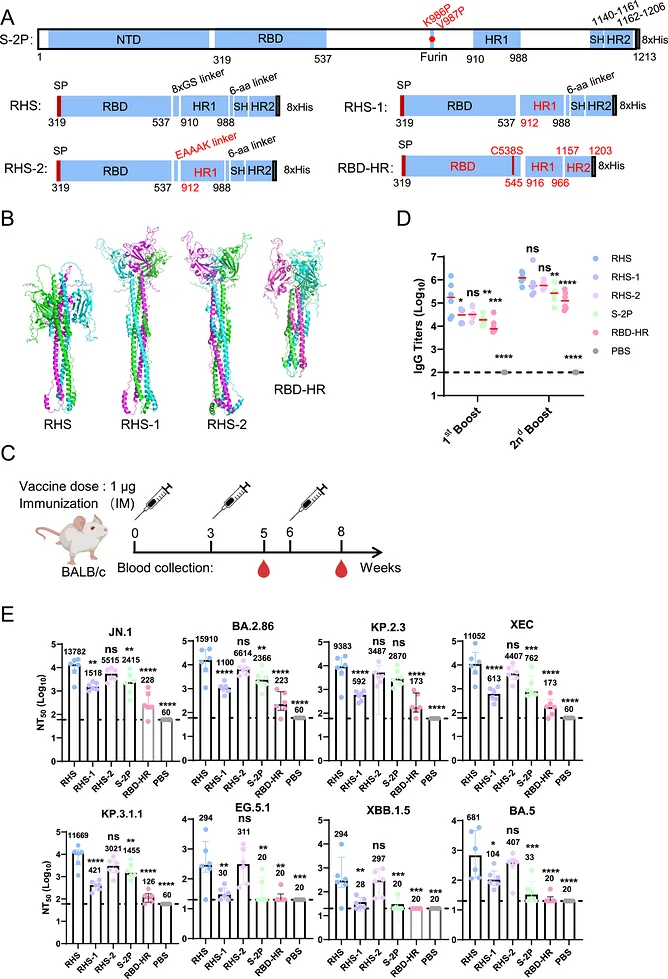
The linker between RBD and HR1 influencing the immunogenicity of RHS antigen. (A) The schematic diagram of JN.1 S‐2P, RHS, RHS‐1, RHS‐2, and RBD‐HR constructs. (B) The structure of RHS, RHS‐1, RHS‐2, and RBD‐HR predicted by AlphaFold3. (C) Immunization schedule. A 6–8‐week‐old female BALB/c mice were also immunized intramuscularly with three doses of 1 µg RHS, RHS‐1, RHS‐2, RBD‐HR, or S‐2P formulated with AddaVax adjuvant. An equal volume of PBS was administered to control mice. The bloods were collected 2 weeks after first and second boost immunization for determining the antibody response. (D) JN.1 RBD‐specific IgG antibody response in RHS, RHS‐1, RHS‐2, RBD‐HR, and S‐2P groups determined by ELISA after first and boost immunization. Median ± IQR are shown (*n* = 6). (E) The NT_50_ were determined by SARS‐CoV‐2 Omicron subvariants JN.1, BA.2.86, KP.2.3, XEC, KP.3.1.1, BA.5, XBB.1.5, and EG.5.1 pseudotyped viruses after last immunization. Median ± IQR are shown (*n* = 6). Animal experiments were repeated at least twice independently with similar results, and data from one representative experiment are shown. ELISA and pseudovirus neutralization assays were performed using duplicate technical wells for each serum sample. Statistical comparisons were performed by one‐way ANOVA with Tukey's multiple‐comparison test (D,E). *p < 0.05; **p < 0.01; ***p < 0.001; ****p < 0.0001; ns, no significant difference.

Linker design can strongly influence the conformation, domain accessibility, and biological function of fusion proteins [24]. In RHS, the 8 × GS linker was designed as a flexible spacer to facilitate dynamic exposure of the RBD for immune recognition. A previous study reported that a Delta variant RBD‐HR trimer vaccine elicited high titers of NAbs [25], in which an 18‐aa C‐terminal segment of RBD functioned as a rigid spacer to the HR rigid‐body scaffold. To evaluate whether the linker configuration between the RBD and HR affects the antigenicity, we constructed two variants: RHS‐1, generated by deleting the 8 × GS linker and two N‐terminal aa of HR1, and RHS‐2, generated by introducing a rigid EAAAK linker between the RBD and HR1 on the RHS‐1 scaffold (Figure 4A). For comparison, we also constructed a JN.1 RBD‐HR plasmid following the same design strategy as the Delta variant RBD‐HR trimer, as well as a prefusion‐stabilized JN.1 S (S‐2P) with K986P, V987P mutations. These constructs were expressed in Expi293F cells and purified by a TALON superflow metal affinity chromatography followed by SEC. SEC and SDS‐PAGE analysis showed that RHS‐1, RHS‐2, and RBD‐HR formed trimeric protein (Figure S6A). AlphaFold 3 modeling predicted that RHS‐1, RHS‐2, and RBD‐HR adopt stable trimeric conformations with rigidly displayed RBD domains (Figure 4B). A 6–8‐week‐old female BALB/c mice were individually immunized with three doses of 1 µg of each antigen (Figure 4C). The sera collected at Weeks 5 and 8 after first and second boost immunization was analyzed by ELISA. At Week 5, RHS elicited significantly higher IgG titers than RHS‐1, S‐2P, and RBD‐HR (Figure 4D), while differences between RHS and RHS‐2 were not significant. At Week 8, IgG titers were comparable among RHS, RHS‐1, and RHS‐2, but remained higher than S‐2P and RBD‐HR (Figure 4D). Neutralization assays revealed that RHS elicited significantly higher NAbs than RHS‐1, S‐2P and RBD‐HR against SARS‐CoV‐2 Omicron subvariants JN.1, BA.2.86, KP.2.3, XEC, KP.3.1.1, EG.5.1, XBB.1.5, and BA.5 (Figure 4E). In contrast, the difference between RHS and RHS‐2 was not significant. To further explore the structural basis for the immunogenicity differences among RHS, RHS‐1, and RHS‐2, we performed negative‐stain electron microscopy (EM) and two‐dimensional (2D) classification analyses. However, no obvious structural differences were detected among these constructs (Figure S6B). We further analyzed the solvent‐accessible surface area (SASA) and spatial accessibility of the displayed RBD (Table S1). SASA analysis showed that RHS had the highest RBD exposure and accessibility, consistent with its superior neutralizing antibody responses. In contrast, RHS‐1 exhibited the greatest reduction in RBD exposure and the strongest spatial hindrance, which may explain its reduced immunogenicity. RHS‐2 showed an intermediate structural profile, with partial reduction in RBD exposure but preservation of immune responses comparable to RHS. Together, these findings suggest that appropriate linker and spatial geometry may contribute to RBD accessibility and the immunogenicity of the RHS scaffold.

### 2.5 RHS Vaccine Induces Potent IgA and IgG Antibody Response in Mucosal Route

**FIGURE 5:**
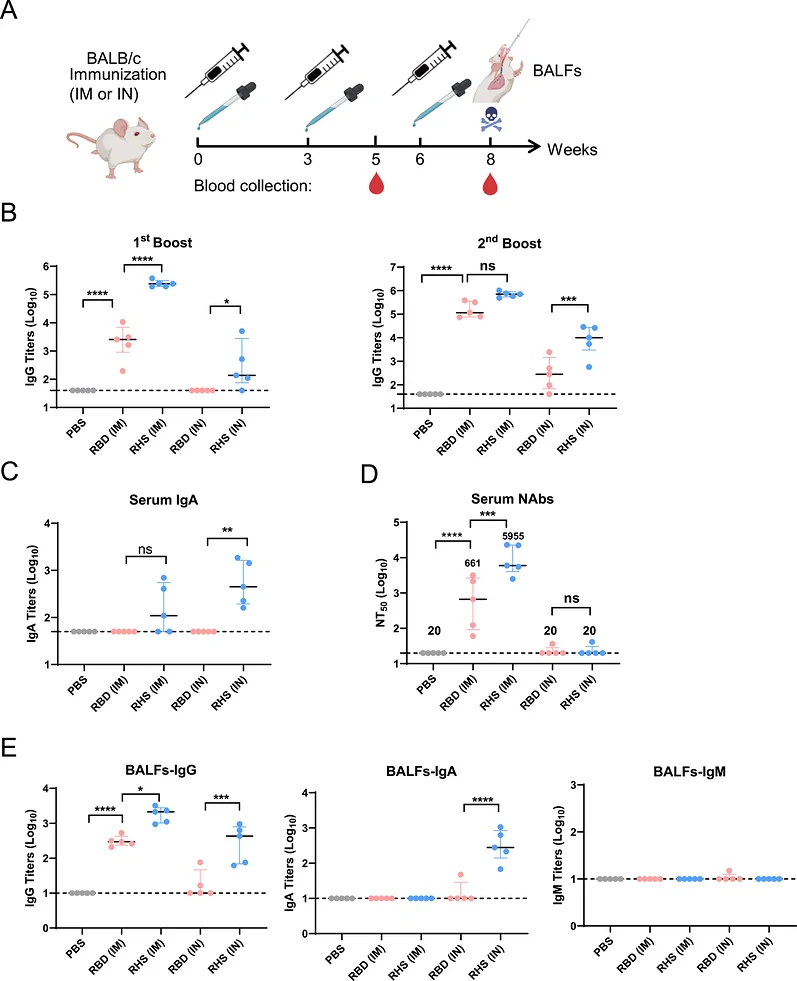
The mucosal immunogenicity of RHS in mice. (A) Immunization schedule. A 6–8‐week‐old female BALB/c mice were immunized intranasally with three doses of 5 µg JN.1 RBD or RHS formulated with CpG adjuvant. A 6–8‐week‐old female BALB/c mice were also immunized intramuscularly with 1 µg RHS or RBD formulated with AddaVax adjuvant as control. An equal volume of PBS was administered to control mice. The blood samples were collected 2 weeks after first and second boost immunization for determining the antibody response. At Week 8, the mice were sacrificed and BALFs were collected for antibody response analysis. (B) JN.1 S‐specific IgG antibody response by IM or IN immunization at Weeks 5 and 8 were determined by ELISA after first and second boosts. Median ± IQR are shown (*n* = 5). (C) Serum JN.1 S‐specific IgA antibody response at Week 8 determined by ELISA. Median ± IQR are shown (*n* = 5). (D) The NT_50_ were determined by SARS‐CoV‐2 Omicron JN.1 variant pseudotyped viruses after last immunization. (E) IgA, IgG, and IgM antibody responses in BALFs determined by ELISA at Week 8. Median ± IQR are shown (*n* = 5). Animal experiments were repeated at least twice independently with similar results, and data from one representative experiment are shown. ELISA and pseudovirus neutralization assays were performed using duplicate technical wells for each serum sample. Statistical comparisons were performed by one‐way ANOVA with Tukey's multiple‐comparison test (B–E). *p < 0.05; **p < 0.01; ***p < 0.001; ****p < 0.0001; ns, no significant difference.

Respiratory mucosa is the primary entry site for the respiratory pathogen [26, 27], and establishing local immunity is essential for protection. To evaluate the mucosal immunogenicity of RHS, 6–8‐week‐old female BALB/c mice received intranasal (IN) immunization with 5 µg JN.1 RBD or RHS plus 10 µg CpG adjuvant (Figure 5A). As a comparison, mice received IM immunization with 1 µg RHS or RBD formulated with AddaVax. Sera were collected at Weeks 5 and 8 after the first and second boosts, and bronchoalveolar lavage fluids (BALFs) were collected at Week 8. RHS elicited higher serum IgG titers than RBD after both IN and IM immunization at Weeks 5 and 8 (Figure 5B). Serum IgA was undetectable after IN immunization with JN.1 RBD but was robustly induced by RHS. In contrast, IM immunization of either antigen failed to elicit detectable IgA (Figure 5C). IM delivery of RHS induced significantly stronger neutralization than RBD, whereas serum NAbs were undetectable following IN immunization with either antigen (Figure 5D). In BALFs, IN delivery of RHS induced potent JN.1 S‐specific IgA and IgG responses, while IgM remained undetectable (Figure 5E). IM delivery of RHS produced strong BALFs IgG but negligible IgA or IgM in BALFs. Together, these results demonstrate that IN delivery of RHS elicits strong mucosal IgA and IgG responses.

### 2.6 Safety Evaluation of RHS Vaccine In Vitro and In Vivo

To assess the in vitro cytotoxicity of RHS, HEK293T cells were incubated with 10 or 100 µg/mL of RBD or RHS proteins (Figure S7A). Neither RHS nor RBD affected cell viability under these conditions. To further evaluate in vivo safety, female BALB/c mice were immunized with 10 µg of RBD or RHS formulated with AddaVax adjuvant. Body weight was monitored for 6 days post‐immunization. No significant changes were observed between groups (Figure S7B). To assess potential tissue toxicity, major organs, including heart, liver, and kidney, were collected after immunization and subjected to histological examination. No detectable pathological alterations were observed in RHS‐immunized mice, indicating a favorable safety profile (Figure S7C–E). Serum biochemical parameters, including alanine aminotransferase (ALT), aspartate aminotransferase (AST), alkaline phosphatase (ALP), urea, and creatinine (CREA), were measured at 0.25, 1, and 6 days post‐immunization (Figure S7F). No significant differences were observed between groups, further supporting the safety profile of the RHS.

### 2.7 RHS Vaccine Elicits Protective Immunity in K18‐hACE2 Mice Challenged With Different Variants of Omicron

To evaluate the protective efficacy of RHS vaccine against the antigen‐matched JN.1 and the antigen‐mismatched EG.5.1, 6–8‐week‐old female K18‐hACE2 mice were immunized with three doses of 10 µg JN.1 RBD or RHS formulated with AddaVax adjuvant on Days 0, 21, and 42 (Figure 6A). On Day 56, the mice vaccinated with RHS elicited significantly higher NAbs against both SARS‐CoV‐2 Omicron JN.1 and EG.5.1 variants compared with the RBD and PBS groups (Figure 6B). On Day 63, mice were randomly divided into two cohorts and challenged intranasally with 10^5^ plaque forming units (PFU) of either SARS‐CoV‐2 Omicron JN.1 or EG.5.1 variant. Lung tissues were collected 3 days post‐challenge for genomic RNA (gRNA) quantification and histopathological analysis. Quantitative reverse transcription‐polymerase chain reaction (qRT‐PCR) analysis showed that RHS vaccination significantly reduced both SARS‐CoV‐2 Omicron JN.1 and EG.5.1 variants gRNA copies compared with the RBD and PBS control groups (Figure 6C). The viral titer in SARS‐CoV‐2 Omicron EG.5.1 variant challenged mice was examined. The results showed that RHS could efficiently reduce the viral titer in lung than RBD group (Figure 6D).

FIGURE 6The protective efficacy of RHS in K18‐hACE2 mice challenged with different Omicron variants. (A) The scheme of vaccine immunization and challenge schedule using SARS‐CoV‐2 Omicron JN.1 or EG.5.1 variants in K18‐hACE2 mice. A 6–8‐week‐old female K18‐hACE2 mice were immunized intramuscularly three doses of 10 µg JN.1 RBD or RHS with AddaVax adjuvant. An equal volume of PBS was administered to control mice. At Day 56, the blood samples were collected determining NAbs. At Day 63, the K18‐hACE2 mice were challenged with 10^5^ PFU SARS‐CoV‐2 Omicron JN.1 or EG.5.1 variants. Lung tissues were collected 3 days post‐challenge for viral load quantification and histopathological analysis. (B) The NT_50_ were determined by SARS‐CoV‐2 Omicron JN.1 and SARS‐CoV‐2 Omicron EG.5.1 variants pseudotyped viruses at Day 56. Median ± IQR are shown (*n* = 10). (C) qRT‐PCR analysis of SARS‐CoV‐2 Omicron JN.1 and EG.5.1 variants genome copies in lung tissues. Mean ± SD are shown (*n* = 5). (D) Viral titer in lungs of mice challenged with SARS‐CoV‐2 Omicron EG.5.1 variants determined by a focus‐forming assay (FFA) and shown as focus‐forming units (FFU) per gram of lung tissue. Median ± IQR are shown (*n* = 5). (E,F) Hematoxylin and eosin (H&E) staining of lung tissue sections from mice challenged with 10^5^ PFU SARS‐CoV‐2 Omicron JN.1 (E) or EG.5.1 (F) variants. (G) Lung pathological scores of mice following JN.1 or EG.5.1 challenge. Data points represent mean ± SD (*n* = 5). (H,I) Immunohistochemistry (IHC) staining of lung tissue sections from mice challenged with SARS‐CoV‐2 Omicron JN.1 (H) or EG.5.1 (I) variants. Scale bar, 50 µm. Animal experiments were repeated at least twice independently with similar results, and data from one representative experiment are shown. Pseudovirus neutralization assays were performed using duplicate technical wells for each serum sample. qRT‐PCR measurements were performed using three technical wells. Statistical comparisons were performed by one‐way ANOVA with Tukey's multiple‐comparison test (B–D, G). *p < 0.05; ***p < 0.001; ****p < 0.0001; ns, no significant difference.

**Figure:**
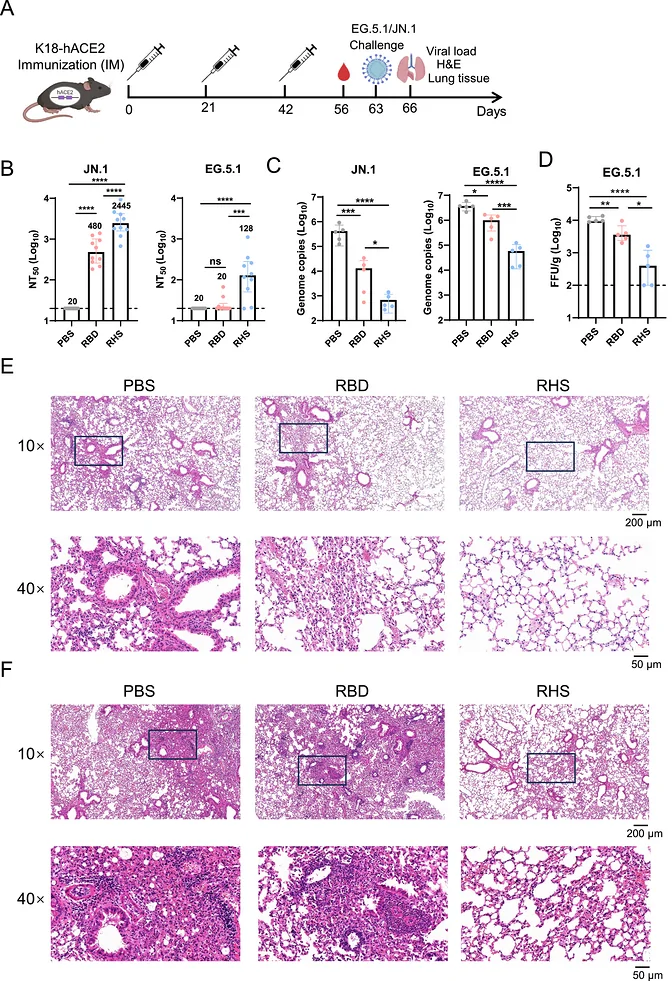

**Figure:**
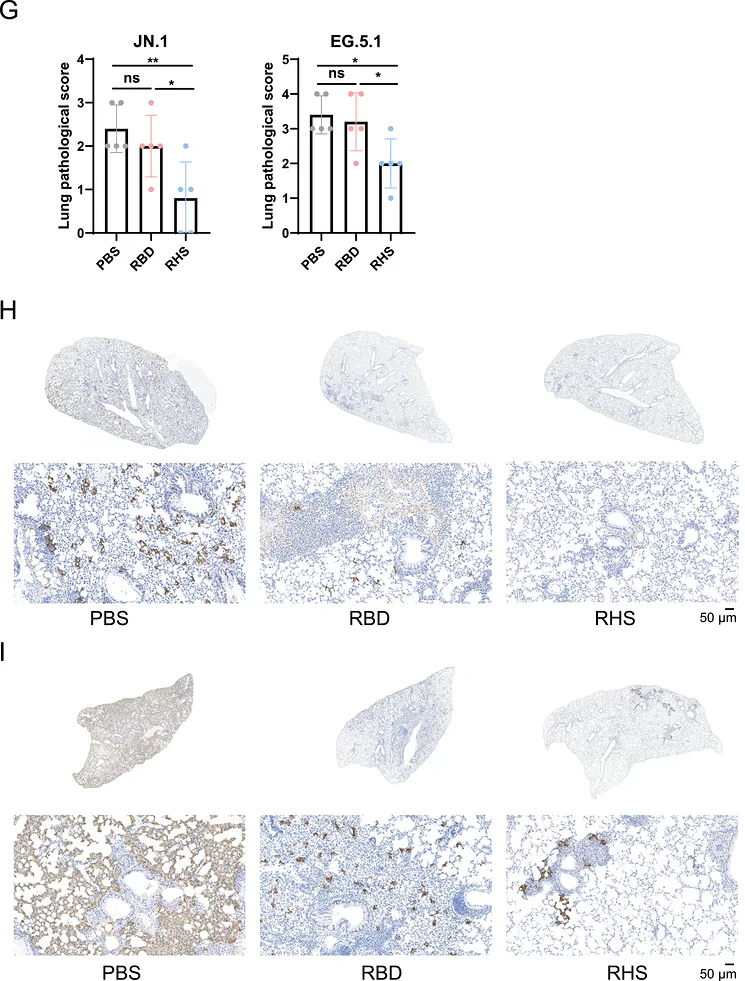

Histopathological and immunohistochemical analyses revealed that PBS‐treated mice challenged with SARS‐CoV‐2 Omicron JN.1 variant exhibited pronounced lung pathology, including thickened alveolar septa, extensive inflammatory cell infiltration, and abundant viral nucleocapsid (N) protein expression (Figure 6E,G,H). RBD‐immunized mice exhibited mild inflammatory infiltration with reduced N protein expression. In contrast, RHS‐immunized mice displayed only minimal lung pathology, lower pathology scores, and no detectable viral antigen expression following SARS‐CoV‐2 Omicron JN.1 variant challenge (Figure 6E,G,H). Notably, SARS‐CoV‐2 Omicron EG.5.1 variant challenge resulted in more severe pulmonary pathology than Omicron JN.1 variant in PBS‐treated mice, characterized by disrupted alveolar architecture, septal thickening, extensive inflammatory infiltration, higher pathology scores, and high levels of N protein expression (Figure 6F,G,I). Severe pulmonary lesions and reduced N protein expression were observed in the RBD‐vaccinated mice. In contrast, RHS vaccinated‐mice maintained well‐preserved alveolar architecture, mild inflammatory cell infiltration, and minimal expression of N protein. These experiments show that RHS confers robust protection against both antigen‐matched and antigen‐mismatched Omicron variants in vivo.

### 2.8 The RHS Antigen Design Can Be Used as a Universal Strategy for the Vaccine Development for SARS‐CoV and MERS‐CoV

The successful development of the SARS‐CoV‐2 RHS vaccine prompted us to generate analogous SARS‐CoV and MERS‐CoV vaccines based on their shared S protein architecture. SARS‐CoV and MERS‐CoV RHS expression plasmids were generated using their corresponding S protein sequences, incorporating the RBD, 8 × GS linker, HR1, 6‐aa linker, SH, HR2, and a C‐terminal 8 × His tag, and the corresponding RBD plasmids were produced as controls (Figure 7A). These constructs were transfected into HEK293T cells or Expi293F cells for protein expression. Western blot, SEC, SDS‐PAGE analyses, and AlphaFold 3 modeling collectively confirmed that both SARS‐CoV and MERS‐CoV RHS proteins self‐assembled into trimers (Figure 7B,C; Figure S8A–D). The purified SARS‐CoV RBD protein was present as monomer and dimer (Figure S8B). To assess whether the RBD in SARS‐CoV RHS recognized hACE2 receptor, we performed ELISA using SARS‐CoV RHS and hACE2‐Fc, with monomeric and dimeric SARS‐CoV RBD serving as controls (Figure 7D; Figure S8B). SARS‐CoV RHS showed stronger binding to hACE2‐Fc than monomeric and dimeric RBD at low doses, with no significant differences at saturating concentrations. MERS‐CoV RHS similarly displayed more robust binding to human dipeptidyl peptidase 4 (hDPP4)‐Fc than monomeric RBD across all tested concentrations (Figure 7E; Figure S8C). To evaluate the immunogenicity of SARS‐CoV and MERS‐CoV RHS protein, 6–8‐week‐old female C57BL/6 mice were intramuscularly immunized with 10 µg SARS‐CoV or MERS‐CoV RHS formulated with alum adjuvant at Weeks 0 and 3 (Figure 7F). Sera of immunized mice were collected at Weeks 2 and 5 for evaluation of antibody response. SARS‐CoV RHS elicited significantly higher SARS‐CoV RBD‐specific IgG antibody than monomeric and dimeric RBD after prime immunization (Figure 7G). After boost immunization, SARS‐CoV RHS also elicited more robust antibody response than monomeric RBD, while the difference was not significant for dimeric RBD. There was no significant difference for binding antibody between MERS‐CoV RHS and RBD after prime immunization (Figure 7H). However, the MERS‐CoV RHS elicited higher titers of binding antibodies than RBD after boost immunization. Pseudovirus neutralization assays showed that both SARS‐CoV and MERS‐CoV RHS induced significantly stronger NAbs than their respective RBDs (Figure 7I,J). Authentic virus neutralization assays were performed to evaluate the neutralization ability of MERS‐CoV RHS vaccine (Figure 7K). Consistently, authentic virus neutralization assays confirmed that MERS‐CoV RHS elicited enhanced neutralizing activity compared with RBD. To explore whether RHS scaffolds could support trimerization of heterologous antigens, we replaced respective RBD with the model antigen ovalbumin (OVA) to generate OVA‐JN.1‐HR1‐SH‐HR2 (HS), OVA‐SARS‐CoV‐HS, and OVA‐MERS‐CoV‐HS (Figure 7L). Following expression in Expi293F cells and purification by TALON affinity chromatography, SDS‐PAGE and SEC analysis confirmed efficient trimerization of all OVA‐HS constructs (Figure 7M; Figure S8E,F). Together, these results demonstrate that the RHS platform is a universal, modular, and adaptable trimerization scaffold suitable for pan‐coronavirus vaccine development and potentially broader antigen design.

FIGURE 7Design and evaluation of SARS‐CoV and MERS‐CoV RHS vaccines. (A) The schematic diagram of SARS‐CoV and MERS‐CoV RHS constructs. (B,C) SEC profiles of SARS‐CoV (B) and MERS‐CoV (C) RHS by a HiLoad 16/600 Superdex 200 pg or Superdex 200 Increase 10/300 GL column. The UV 280 nm absorbance curves are indicated. The SDS‐PAGE analysis of the purified SARS‐CoV (B) and MERS‐CoV (C) RHS protein in non‐denaturing and denaturing conditions were shown on the right of each corresponding SEC. (D) The binding activity of SARS‐CoV monomer and dimer RBD, as well as RHS to hACE2‐Fc at indicated concentration determined by ELISA. Data points represent mean ± SD (*n* = 3) from three independent experiments. (E) The binding activity of MERS‐CoV RBD and RHS to human dipeptidyl peptidase 4 (hDPP4)‐Fc at the indicated concentration determined by ELISA. Data points represent mean ± SD (*n* = 3) from three independent experiments. (F) Immunization schedule. A 6–8‐week‐old female C57BL/6 mice were intramuscularly immunized with two doses of 10 µg SARS‐CoV monomer RBD, dimer RBD, RHS, MERS‐CoV RBD, or RHS, each formulated with alum adjuvant. An equal volume of PBS was administered to control mice. The blood samples were collected two weeks after each immunization for determining the antibody response. (G) SARS‐CoV RBD‐specific IgG antibody response determined by ELISA at Weeks 2 and 5. Median ± IQR are shown (*n* = 5). (H) MERS‐CoV RBD‐specific IgG antibody response determined by ELISA at Weeks 2 and 5. Median ± IQR are shown (*n* = 6). (I) The NT_50_ against SARS‐CoV pseudotyped viruses at Week 5. Median ± IQR are shown (*n* = 5). (J) The NT_50_ against MERS‐CoV pseudotyped viruses at Week 5. Median ± IQR are shown (*n* = 6). (K) FRNT_50_ against authentic MERS‐CoV. Data are presented as median ± IQR (*n* = 6). (L) The schematic diagrams of the OVA‐JN.1‐HS, OVA‐SARS‐CoV‐HS, and OVA‐MERS‐CoV‐HS constructs. These fusion proteins were generated by replacing the RBD in the corresponding SARS‐CoV‐2 JN.1, SARS‐CoV, and MERS‐CoV RHS constructs with OVA. (M) SDS‐PAGE analysis of purified OVA‐JN.1‐HS, OVA‐SARS‐CoV‐HS, and OVA‐MERS‐CoV‐HS fusion protein in non‐denaturing and denaturing conditions. For denaturing conditions, samples were mixed with 5 × SDS‐PAGE protein loading buffer containing β‐mercaptoethanol and heated at 100°C for 10 min prior to SDS‐PAGE analysis. For non‐denaturing conditions, samples were mixed with 5 × SDS‐PAGE protein loading buffer without β‐mercaptoethanol and analyzed without heat treatment. SDS‐PAGE, SEC analysis, and animal experiments were repeated at least twice independently with similar results, and data from one representative experiment are shown. ELISA and pseudovirus neutralization assays were performed using duplicate technical wells for each serum sample. Statistical comparisons were performed by one‐way ANOVA with Tukey's multiple‐comparison test (D, G–K) or unpaired one‐tailed Student's *t*‐test (E). *p < 0.05; **p < 0.01; ***p < 0.001; ****p < 0.0001; ns, no significant difference.

**Figure:**
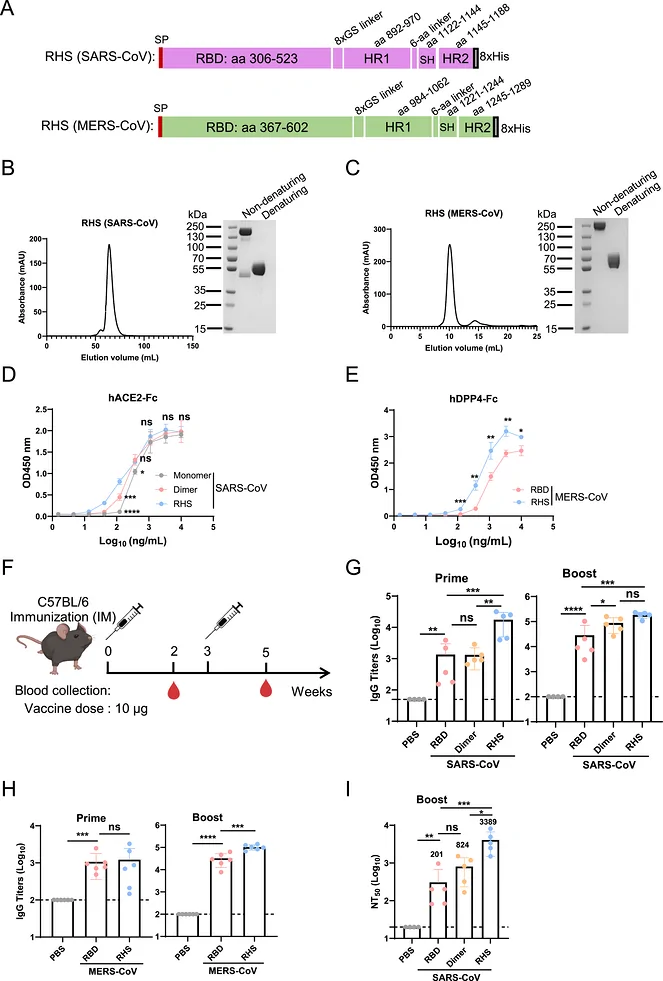

**Figure:**
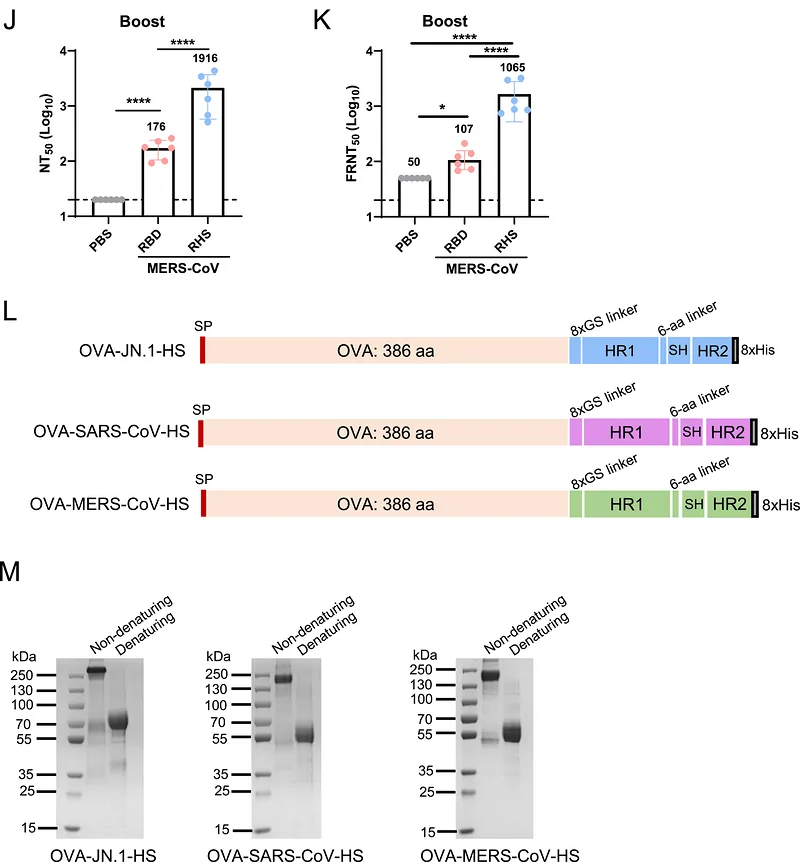

## 3 Discussion

Multiple vaccine platforms have been explored to develop coronavirus vaccines, including adenovirus‐vectored vaccines, mRNA vaccines, inactivated vaccines, live attenuated vaccines, and protein‐based vaccines [28]. Among these, protein‐based vaccines exhibit excellent safety profiles but often suffer from limited immunogenicity. In particular, monomeric RBD‐based vaccine generally exhibit limited [29, 30]. To enhance RBD‐induced antibody responses, exogenous scaffold‐based multimerization strategies have been widely employed [7, 8, 31]. Nevertheless, these foreign elements may raise safety concerns by eliciting off‐target immune responses. The conserved HR1 and HR2 regions of the S2 subunit self‐assemble into a stable 6‐HB [12], providing a natural trimerization mechanism. Consistent with this concept, a previous study reported that an RBD‐HR trimer incorporating a truncated HR1‐HR2 trimer region exhibited promising immunogenicity [25], suggesting that endogenous multimerization domains may enhance antigenicity while avoiding undesired immune responses. However, the high mutation frequency of the RBD poses a major obstacle to the development of broad‐spectrum coronavirus vaccines [14]. Although mosaic RBD nanoparticles can elicit broad immunity [32], their increased production complexity and reliance on exogenous scaffolds remain potential limitations.

Conserved S2 epitopes, including FP, HR1, HR2, and SH, provide an alternative route to broaden coronavirus antibody responses [15]. Vaccine candidates based on HR1‐HR2‐HR1, HR2‐HR1‐HR2, prefusion‐stabilized S2, or FP‐HR1‐HR2 constructs have been shown to elicit broadly NAbs [16, 33, 34, 35]. However, S2‐targeted vaccines generally induce lower NAbs than RBD‐based vaccines, highlighting the potential advantage of combining conserved S2 epitopes with the RBD.

In this study, we rationally designed the RHS fusion protein by integrating the RBD with a native HR1‐SH‐HR2 trimeric scaffold. Our data show that RHS combines broad neutralizing activity, T‐cell responses, and in vivo protection in a single trimeric antigen design. Importantly, incorporation of the conserved SH epitope induced pan‐β‐coronaviruses SH‐specific antibodies, substantially enhancing both the breadth and magnitude of the immune response. Together, these findings establish RHS as a novel antigen‐engineering strategy with strong potential for broad‐spectrum coronavirus vaccine development.

A previous study demonstrated that a C‐terminal 18‐aa segment of the RBD can function as a rigid linker in Delta strain RBD‐HR trimer, resulting in high neutralizing antibody titers [25]. In contrast, the JN.1 RBD‐HR trimer in our expression system failed to elicit strong neutralizing responses. Several factors may explain this difference. First, sequence alignment revealed 28‐aa substitutions within the RBD between the Delta strain and the Omicron JN.1 variant, reflecting substantial antigenic evolution. These mutations may alter the antigenic surface and affect antibody recognition, thereby influencing immunogenicity. Second, differences in protein expression systems may also contribute. The previous study used an insect cell expression system, whereas our proteins were produced in mammalian cells. Such differences can affect glycosylation patterns, protein folding, and overall antigen conformation, all of which may influence the immunogenic properties of the RBD‐HR construct. Further studies directly comparing JN.1 RBD‐HR proteins produced in different expression systems will be required to clarify this issue.

Our data further suggest that linker design contributes to the immunogenicity of the RHS scaffold. In the RHS design, complete removal of the flexible linker between the RBD and HR1 markedly impaired immunogenicity, whereas inclusion of either flexible or rigid linkers preserved robust immune responses. Consistent with these findings, SASA analysis indicated that RHS and RHS‐2, which contain flexible and rigid linkers, respectively, maintained higher RBD exposure and accessibility than RHS‐1, which lacks the linker. A plausible explanation is that the linker helps maintain appropriate spatial presentation of the RBD, thereby improving its accessibility to BCR and antibodies. In contrast, linker deletion may impose steric constraints that partially occlude the RBD and reduce immune recognition. These data suggest that linker design and spatial geometry are important determinants of immunogenicity in fusion protein‐based vaccine design. However, the negative‐stain TEM and 2D classification analyses provided only limited structural resolution, and the current structural interpretation is mainly based on computational modeling and SASA analysis. Therefore, the proposed mechanism remains inferential. High‐resolution structural approaches, such as cryo‐EM analysis, will be required to elucidate how linker architecture influences antigen presentation and immune responses at the molecular level.

Human coronaviruses encode a type I transmembrane S glycoprotein, in which the conserved HR1 and HR2 regions assemble into a 6‐HB during viral entry [13]. Consistent with the functional importance of this region, the HR1‐targeting fusion inhibitor EK1 exhibits broad‐spectrum antiviral activity [13, 17]. Leveraging this shared structural feature, we extended the RHS platform to SARS‐CoV and MERS‐CoV vaccine design. Both SARS‐CoV and MERS‐CoV RHS vaccines were successfully constructed and exhibited strong immunogenicity. Given the conserved HR1‐HR2 interaction shared among human coronaviruses [13, 36], the RHS platform has the potential to be extended to other coronaviruses, including OC43, 229E, and NL63. However, OC43‐, 229E‐, and NL63‐based RHS constructs have not yet been generated, and their immunogenicity, neutralizing activity, and protective efficacy remain to be determined. Furthermore, substitution of the RBD with a heterologous OVA antigen demonstrated that the HR1‐SH‐HR2 scaffold efficiently drives trimerization of non‐coronavirus antigens. It indicates that this platform is not restricted to coronavirus RBD display. Such modularity provides a basis for the development of multivalent, mosaic, or pathogen‐adaptable subunit vaccines using a shared endogenous trimerization framework. Beyond protein subunit vaccines, the modular RHS antigen design may also be applicable to other vaccine platforms, including mRNA and adenoviral vector vaccines, thereby broadening its translational potential. Together, these findings establish RHS as a versatile and broadly applicable platform for subunit vaccine development.

In this study, three adjuvants were selected to examine RHS immunogenicity under different vaccination conditions. For IM immunization, AddaVax and alum are widely used in protein subunit vaccine development [37, 38]. Under both AddaVax‐ and alum‐adjuvanted conditions, RHS consistently elicited stronger neutralizing antibody responses than RBD. Mucosal immunity was additionally assessed using CpG, a Toll‐like receptor 9 agonist known to promote IgA responses [39]. Similarly, CpG‐adjuvanted RHS induced stronger mucosal IgA and IgG responses than RBD. These findings suggest that the enhanced immunogenicity of RHS is largely attributable to the antigen design itself rather than a specific adjuvant formulation. Nevertheless, the use of different adjuvants may have influenced the magnitude and quality of the immune responses observed, and the absence of a unified adjuvant control limits direct comparisons across vaccine platforms and immunization routes. Future studies employing a standardized adjuvant system will help further define the relative contributions of antigen design and adjuvant‐mediated immune modulation. Although CpG‐adjuvanted RHS induced robust mucosal IgA responses, serum neutralizing antibody titers remained limited. These results suggest that further optimization of mucosal adjuvants will be required to improve neutralizing responses. Candidate mucosal adjuvants, including cholera toxin B (CTB) subunit, the heat‐labile enterotoxin B subunit of *Escherichia coli* (LTB), and flagellin, should therefore be explored in future studies [40]. The protective efficacy of IN RHS vaccination was not evaluated in this study. Accordingly, lung viral load quantification and histopathological analyses were not performed following IN immunization. Future challenge studies will be necessary to determine whether the enhanced mucosal immune responses induced by RHS translate into protection against infection and disease.

This study has several limitations. Although the RHS vaccine induced potent NAbs against Omicron variants, it showed comparatively reduced neutralizing activity against the ancestral‐like D614G variant, which is antigenically similar to the original Wuhan strain. This may reflect the fact that RHS was designed based on the Omicron JN.1 variant, which has undergone substantial antigenic drift relative to ancestral SARS‐CoV‐2 strains. Consequently, RHS may preferentially elicit antibodies targeting Omicron‐adapted RBD epitopes, while inducing only limited cross‐reactive RBD‐directed responses against ancestral‐like viruses. In addition, although SH‐elicited antibodies are broadly cross‐reactive, their neutralizing potency in vaccine appears to be relatively modest [41]. Therefore, cross‐neutralization against D614G variant is likely mediated by the combined contribution of cross‐reactive RBD‐directed and SH‐specific antibodies, rather than by either component alone. Another limitation of this study is that the neutralizing activity of the SARS‐CoV RHS vaccine was evaluated only using a pseudovirus neutralization assay because authentic SARS‐CoV was not available for testing. Similarly, neutralization against the recently emerged SARS‐CoV‐2 Omicron variants XFG and NB.1.8.1 was assessed using pseudovirus‐based assays. Although pseudovirus neutralization assays are widely used and provide a safe and reliable approach for evaluating antibody‐mediated neutralization, they do not fully recapitulate the complexity of authentic viral infection and therefore cannot directly demonstrate protection in vivo. In the present study, the neutralization trends observed in pseudovirus assays were consistent with those obtained using authentic Omicron JN.1, Omicron EG.5.1, and MERS‐CoV neutralization assays, supporting the utility of the pseudovirus system for comparative evaluation. Nevertheless, further studies using authentic SARS‐CoV, Omicron XFG, and Omicron NB.1.8.1 viruses will be required to validate the breadth and protective potential of RHS‐induced antibody responses. In addition, the immunogenicity of RHS was evaluated in immunocompetent BALB/c and C57BL/6 mice, whereas its protective efficacy was assessed in K18‐hACE2 mice. BALB/c and C57BL/6 mice are useful for evaluating vaccine‐induced humoral, cellular, and mucosal immune responses, but they do not support productive SARS‐CoV‐2 infection because murine ACE2 does not efficiently mediate viral entry. In contrast, K18‐hACE2 mice express human ACE2 and are susceptible to SARS‐CoV‐2 infection, making them suitable for viral challenge studies. However, mouse models alone cannot fully recapitulate human infection, disease pathogenesis, or immune responses. Further studies in non‐human primates will therefore be necessary to more comprehensively assess the immunogenicity, protective efficacy, and safety of this vaccine strategy, followed before eventual evaluation in human clinical trials.

Immune imprinting remains a major challenge for vaccine development, particularly for SARS‐CoV‐2. Vaccine strain updating has become a primary mitigation strategy [42]. Because RHS elicits stronger and broader antibody responses than monomeric RBD, RHS‐based updated or multivalent immunogens may help broaden or redirect pre‐existing immunity. Heterologous immunization strategies using RHS constructs may also prove beneficial. However, whether multivalent RHS formulations or alternative delivery regimens can overcome immune imprinting remains to be determined in real‐world vaccination settings.

Future optimization of the RHS platform for clinical translation should include systematic antigen dose‐gradient screening in additional preclinical models, evaluation of heterologous prime‐boost regimens, and optimization of combined adjuvant formulations. In the present study, antigen dose optimization was performed only in BALB/c mice, and systematic dose‐gradient studies should be performed in other animal models. Our previous studies have shown that heterologous prime‐boost strategies can enhance the magnitude and breadth of immune responses [43], and such approaches warrant investigation for RHS‐based vaccines. In addition, combined adjuvant systems, such as MPLA/QS‐21‐containing formulations or alum plus CpG, may further improve humoral and cellular immunity. Multivalent or chimeric RHS antigen designs incorporating conserved B‐cell and T‐cell epitopes or antigenic components from different coronaviruses may also help broaden protection against diverse coronavirus lineages. The present study provides proof‐of‐concept evidence for the RHS platform in mouse models. Before clinical translation, further evaluation in non‐human primates will be required to assess immunogenicity, safety, and protective efficacy, antigen dose selection, and translational potential under more clinically relevant conditions. Favorable outcomes from these studies could support subsequent evaluation of the RHS platform in early‐phase clinical trials.

In conclusion, this work establishes RHS as a potent, modular vaccine platform that exploits endogenous viral elements to drive trimerization and incorporate conserved epitopes such as the SH. By eliciting broad and protective immunity against highly evasive variants, RHS provides a promising pathway toward universal coronavirus vaccine development and strengthens preparedness against future coronavirus pandemics.

## 4 Experimental Section/Methods

### 4.1 Ethics Statement

All experimental procedures were approved by the institutional animal care and use committee of Guangzhou National Laboratory (ethics number: GZLAB‐AUCP‐2024‐04‐A13). SARS‐CoV‐2 Omicron variants challenge experiment was performed in biosafety Level 3 (BSL‐3) laboratory of Guangzhou National Laboratory.

### 4.2 Cell Lines and Antibodies

HEK293T (ATCC CRL‐11268) and Vero‐E6 (ATCC CRL‐1586) cells were cultured in Dulbecco's modified Eagle medium (DMEM) (Gibco), supplemented with 10% fetal bovine serum (FBS) (ExCell Bio) and 1× penicillin‐streptomycin (Corning). Expi293F cells were purchased from OPM Biosciences and cultured in 293F Hi‐exp Medium (OPM Biosciences). Anti‐His tag antibody (Cat. No. 105327‐MM02T) was purchased from Sino Biological. Anti‐His tag horseradish peroxidase (HRP)‐conjugated antibody (Cat. No. HRP‐66005) was purchased from Proteintech.

### 4.3 Plasmid Construction

Codon‐optimized S genes containing a 19‐aa deletion at the C‐terminus of SARS‐CoV‐2 Omicron BA.2.86 (GISAID: EPI_ISL_18110065) and XBB.1.5 (GISAID: EPI_ISL_12568880) were synthesized (Tsingke) and inserted into the pcDNA3.4 vector to generate pcDNA3.4‐BA.2.86‐S and pcDNA3.4‐XBB.1.5‐S, respectively. The pcDNA3.4‐BA.2.86‐S plasmid was used as the parental backbone for the generation of multiple Omicron subvariant S expression plasmids by PCR‐based site‐directed mutagenesis. Briefly, mutagenic primers carrying the desired nucleotide substitutions or deletions were designed to flank the target mutation sites. PCR amplification was performed using the parental plasmid as the template, and the amplified products were ligated to generate circular mutant plasmids. The ligation plasmids were transformed into *E. coli* DH5α competent cells. Individual colonies were screened by colony PCR, and all positive plasmids were verified by Sanger sequencing across the mutated regions. Using pcDNA3.4‐BA.2.86‐S as the template, the following BA.2.86‐derived Omicron subvariant S expression plasmids were generated: pcDNA3.4‐JN.1‐S (L455S); pcDNA3.4‐SLip‐S (L455S + F456L); pcDNA3.4‐KP.2‐S (R346T + L455S + F456L + V1104L); pcDNA3.4‐KP.3‐S (L455S + F456L + Q493E + V1104L); pcDNA3.4‐KP.2.3‐S (ΔS31 + H146Q + R346T + L455S + F456L + V1104L); pcDNA3.4‐KP.3.1.1‐S (ΔS31 + L455S + F456L + Q493E + V1104L); pcDNA3.4‐XEC‐S (T22N + F59S + L455S + F456L + Q493E + V1104L). The pcDNA3.4‐JN.1‐S‐2P plasmid was generated from pcDNA3.4‐JN.1‐S by introducing the furin cleavage site mutations (R679A and R681A) and the prefusion‐stabilizing 2P mutations (K982P and V983P). To produce a soluble JN.1 S‐2P expression construct, the transmembrane domain and cytoplasmic tail region, corresponding to aa 1214‐1273 of D414G‐S, were deleted, and a C‐terminal 8 × His tag was added from pcDNA3.4‐JN.1‐S. The pcDNA3.4‐EG.5.1‐S plasmid was generated from the pcDNA3.4‐XBB.1.5‐S construct by introducing Q52H and F456L using the same PCR‐based mutagenesis strategy. The XFG and NB.1.8.1 S expression plasmids were kindly provided by Dr. Yunlong Cao from Peking University. S expression plasmids for SARS‐CoV‐2 D614G (GISAID: EPI_ISL_422425), BA.5 (GISAID: EPI_ISL_11542604), SARS‐CoV (GenBank: AY864805.1), and MERS‐CoV (GenBank: JX869059.2) had been previously generated in our laboratory.

The SARS‐CoV‐2 RHS expression construct was designed by sequentially fusing the following elements from the N‐ to C‐terminus: the Igκ signal peptide, JN.1 RBD (aa 319‐537), an 8 × GS linker, HR1 (aa 910‐988), a 6‐aa linker (SGGRGG), the SH (aa 1140‐1161), HR2 (aa 1162‐1206), and a C‐terminal 8 × His tag. The full‐length coding sequence was synthesized and cloned into pcDNA3.4 to generate pcDNA3.4‐SARS‐CoV‐2‐RHS. The JN.1 RBD‐HR construct was designed by fusing the Igκ signal peptide, JN.1 RBD (aa 320‐545) containing the C534S mutation, HR1 (aa 916‐966), HR2 (aa 1157‐1203), and a C‐terminal 8 × His tag, followed by synthesis and cloning into pcDNA3.4 to generate pcDNA3.4‐JN.1‐RBD‐HR. The SARS‐CoV RHS and MERS‐CoV RHS plasmids were constructed using the same modular design strategy. The SARS‐CoV RHS construct consisted of the Igκ signal peptide, RBD (aa 306‐523), an 8 × GS linker, HR1 (aa 892‐970), the SGGRGG linker, SH (aa 1122‐1144), HR2 (aa 1145‐1188), and an 8 × His tag. The MERS‐CoV RHS construct consisted of the Igκ signal peptide, RBD (aa 367‐602), an 8 × GS linker, HR1 (aa 984‐1062), the SGGRGG linker, SH (aa 1221‐1244), HR2 (aa 1245‐1289), and an 8 × His tag. The corresponding coding sequences were synthesized and cloned into pcDNA3.4 to generate pcDNA3.4‐SARS‐CoV‐RHS and pcDNA3.4‐MERS‐CoV‐RHS, respectively. Additional RHS‐derived constructs were generated by PCR amplification using the corresponding RHS plasmids as templates. For the monomeric RBD constructs, the coding regions corresponding to JN.1 RBD, SARS‐CoV RBD, or MERS‐CoV RBD were amplified together with the Igκ signal peptide and a C‐terminal 8 × His tag, and cloned into pcDNA3.4 to generate pcDNA3.4‐JN.1‐RBD, pcDNA3.4‐SARS‐CoV‐RBD, and pcDNA3.4‐MERS‐CoV‐RBD, respectively. The RHS deletion constructs were generated from pcDNA3.4‐SARS‐CoV‐2‐RHS by PCR amplification excluding the indicated regions. pcDNA3.4‐RHSΔSH was constructed by deleting the SH‐coding sequence while retaining the remaining RHS elements. pcDNA3.4‐RHSΔ6‐aa was constructed by deleting the 6‐aa SGGRGG linker between HR1 and SH. pcDNA3.4‐RHS‐1 was generated by deleting the 8 × GS linker between RBD and HR1 together with the first two aa of HR1, thereby producing a junction. pcDNA3.4‐RHS‐2 was generated using pcDNA3.4‐RHS‐1 as the template by PCR amplification with primers encoding the rigid 5‐aa linker EAAAK, which was inserted at the RBD‐HR1 junction.

The OVA‐HS constructs were generated by replacing the RBD‐coding region in the corresponding RHS plasmids with the OVA coding sequence. Specifically, the RBD regions in pcDNA3.4‐SARS‐CoV‐2‐RHS, pcDNA3.4‐SARS‐CoV‐RHS, and pcDNA3.4‐MERS‐CoV‐RHS were removed by PCR amplification, and the OVA was introduced by homologous recombination using the ClonExpress recombination kit (Vazyme, C113‐02), generating pcDNA3.4‐OVA‐JN.1‐HS, pcDNA3.4‐OVA‐SARS‐CoV‐HS, and pcDNA3.4‐OVA‐MERS‐CoV‐HS, respectively. All mutation‐ or recombination‐derived plasmids were verified by Sanger sequencing before use.

### 4.4 Protein Expression and Purification

His‐tagged antigen proteins were produced by transient transfection of Expi293F cells with plasmids and polyethyleneimine (PEI) at a DNA‐to‐PEI ratio of 1:4. The culture medium was harvested at 5 days post‐transfection by centrifugation at 10 000 × *g* for 1 h at 4°C. After clarification, the supernatant was loaded onto a TALON superflow metal affinity resin. Resin‐bound protein was eluted with buffer containing 50 mM Tris‐HCl, 300 mM NaCl, and 150 mM imidazole. The eluate was further purified by SEC using a HiLoad 16/600 Superdex 200 pg column or Superdex 200 Increase 10/300 GL column pre‐equilibrated with PBS. The eluted protein was concentrated in ultracentrifugal filter and stored at −80°C until use. SDS‐PAGE and SEC were used to determine protein purity. Only preparations with a purity greater than 90% were used for animal immunization experiments. Endotoxin levels of purified RHS were measured using a ToxinSensor Chromogenic Limulus amebocyte lysate (LAL) Endotoxin Assay Kit (GenScript, L00350) and was < 0.05 EU per µg. To ensure consistency in antigen quality, SARS‐CoV RHS and MERS‐CoV RHS proteins were expressed, purified, and quality‐controlled using the same protocol as SARS‐CoV‐2 RHS.

### 4.5 Antibody Expression and Purification

The heavy‐ and light‐ chain genes of S2P6, CV3‐25, CC99.103, and CB6 antibodies was cloned in pCMV3 expression vector. For each antibody, matched heavy‐ and light‐chain plasmids were introduced into Expi293F cells at a 1:1 molar ratio. The culture supernatants of Expi293F cells were harvested five days after transfection by centrifugation at 10 000 × *g* for 1 h at 4°C. The supernatants were loaded onto a protein A agarose column. The eluted antibodies were concentrated and exchanged with PBS buffer using 50 kDa molecular weight cut‐off MWCO Amicon Ultra filtration device (Merck Millipore).

### 4.6 SDS‐PAGE and Coomassie Brilliant Blue Staining

Purified protein or cell supernatants were mixed with 5 × SDS‐PAGE protein loading with (denaturing conditions) or without β‐mercaptoethanol (non‐denaturing conditions). For denaturing samples, the mixture was boiled at 100°C for 10 min, then briefly centrifuged for 10 s. Both denaturing and non‐denaturing samples were loaded onto 4%–20% precast gels (GenScript) and separated by electrophoresis according to the manufacturer's instructions. After completion of electrophoresis, the SDS‐PAGE gel was removed from the glass plates and briefly rinsed with double‐distilled water (ddH_2_O). The gel was heated in ddH_2_O at 100°C for 5 min, followed by staining with Coomassie Brilliant Blue fast staining solution for 10 min with gentle agitation. The gel was then destained in ddH_2_O with continuous agitation until protein bands were clearly visible. Finally, the gel was imaged and analyzed.

### 4.7 Analytical Ultracentrifugation

Samples were prepared at an absorbance of 0.6–0.8 at UV 280 nm and loaded into standard two‐sector Epon centerpieces. Experiments were conducted using an Optima AUC analytical ultracentrifuge (Beckman Coulter, USA) equipped with an An‐60 Ti rotor, operated at 50 000 rpm and 16°C. Data were analyzed using SEDFIT software.

### 4.8 Enzyme‐Linked Immunosorbent Assay

Ninety‐six‐well plates were coated overnight at 4°C with 100 µL per well of JN.1 RBD or S protein (0.5 µg/mL). After the plates were washed twice with PBST (0.05% Tween 20 in 1 × PBS), the plates were blocked for 1 h at room temperature using 5% skimmed milk in PBST. After washed with PBST, heat‐inactivated mouse sera were serially diluted (four‐fold), added to the washed plates, and incubated 1 h at 37°C. Subsequently, the wells of plates were then washed and incubated with an HRP‐conjugated goat anti‐mouse IgG antibody (Beyotime) at room temperature for 1 h. After the final wash, the signals were developed with 100 µL TMB substrate and terminated with 50 µL 2 M sulfuric acid. The OD values at 450 nm (OD450) were measured. Endpoint titers were defined as the reciprocals of the maximal dilutions at which the OD450 values were equal to or higher than the cut‐off value (2.1 times that from the negative control wells).

### 4.9 Animals

A 6–8 weeks female BALB/c and C57BL/6 mice were purchased from Guangdong GemPharmatech Company. K18‐hACE2 mice were purchased from the Jackson Laboratory. All mice were housed in the Animal Center of Guangzhou National Laboratory.

### 4.10 Animal Immunization

To assess the immunogenicity of the RHS construct, female BALB/c mice were intramuscularly immunized with JN.1 RBD or RHS protein at doses of 0.2, 1, or 10 µg, formulated with AddaVax adjuvant (InvivoGen). Immunizations were administered at Weeks 0, 3, and 6. Blood samples were collected at Weeks 2, 5, and 8 for serological analysis. At Week 8, mice were euthanized, and spleens were harvested for cellular immune response assays. For mucosal immunization schedule, the female BALB/c were IN vaccinated with 5 µg JN.1 RBD or RHS protein adjuvanted with CpG. The female BALB/c were also IM vaccinated with 1 µg JN.1RBD or RHS protein adjuvanted with AddaVax as control. Immunizations were administered at Weeks 0, 3, and 6. Blood samples were collected at Weeks 5 and 8 for serological analysis. At Week 8, mice were euthanized, and BALFs were harvested for subsequent analysis. To assess the impact of the linker between RBD and HR1 on immunogenicity, female BALB/c mice were intramuscularly immunized with 1 µg of JN.1 RBD, RHS, RHS‐1, RHS‐2, RBD‐HR, or S‐2P protein formulated with AddaVax. Immunizations were administered at Weeks 0, 3, and 6. Serum samples were collected at Weeks 5 and 8 for subsequent analysis. To assess the contribution of the SH epitope, female C57BL/6 mice were intramuscularly immunized with 10 µg of JN.1 RBD, RHS, or RHSΔSH protein formulated with AddaVax at Weeks 0, 3, and 6. Serum samples were collected at Weeks 2, 5, and 8 for subsequent analysis. To determine the protective efficacy of RHS, female K18‐hACE2 mice were immunized with 10 µg of JN.1 RBD or RHS at Weeks 0, 3, and 6. To evaluate the immunogenicity of SARS‐CoV and MERS‐CoV RHS proteins, C57BL/6 mice were intramuscularly immunized with two doses of 10 µg of SARS‐CoV or MERS‐CoV RBD or RHS protein formulated with alum (InvivoGen). Serum samples were collected at Weeks 2 and 5 for subsequent analysis.

### 4.11 ICS Assay

In brief, lymphocytes were isolated from the spleen by processing through a 100 µm strainer with a syringe plunger. Moreover, 2 × 10^6^ spleen cells/well were re‐suspended in 100 mL of R10 media. Each sample was assessed with mock (100 mL of R10 plus 0.5% DMSO; background control), peptides (2 mg/mL), and Leukocyte Activation Cocktail (diluted 1:200) from BD (100 mL/well) and incubated at 37°C for 1 h. After incubation, 0.25 mL of GolgiPlug in 50 mL of R10 was added to each well and incubated at 37°C for 8 h and then held at 4°C overnight. The next day, the cells were washed twice with MACS buffer, stained with live/dead dye Fixable Viability Stain 780 and Purified Rat Anti‐Mouse CD16/CD32 monoclonal for 10 min and then stained with predetermined titers of monoclonal against CD8a (clone 53–6.7; BUV395), CD3 (clone 17A2; BUV737), CD103 (clone 2E7; BV605), CD44 (clone IM7; BV711), CD62L (clone MEL‐14; Alexa Fluor 488), CD4 (clone RM4–5; PE/Dazzle 594), and CD69 (clone H1.2F3; PE‐Cy7) (all antibodies diluted 1:100) for 1 h. Cells were then washed twice with MACS buffer and incubated with 100 mL of BD Cytofix/CytoPerm Fixation/Permeabilization solution for 15 min. Cells were then washed twice with 1 × Perm Wash buffer (BD Perm/Wash Buffer 10 × in CytoFix/CytoPerm Fixation/Permeabilization kit diluted with MACS buffer and passed through 0.22 mm filter) and stained intracellularly with mAbs against IFN‐γ (clone XMG1.2; BV421), IL‐2 (clone JES6‐5H4; PE), IL‐4 (clone 11B11; APC), and TNF‐α (clone MP6‐XT22; Alexa Fluor 700) (all antibodies diluted 1:100) for 1 h. Cells were then washed twice with 1 × Perm Wash buffer and washed once with MACS buffer, then fixed with 250 µL of freshly prepared 2% formaldehyde. Fixed cells were analyzed by the Agilent NovoCyte Advanteon Flow Cytometer. Data were analyzed using FlowJo v10.8.1.

### 4.12 ELISpot Analysis

In brief, ELISpot plates were coated with anti‐mouse IFN‐γ or IL‐4 capture antibodies (Mabtech) and then incubated at 4°C overnight. Freshly isolated splenic lymphocytes were added at 3 × 10^5^ cells per well and stimulated with a JN.1 S peptide pool (2 µg/mL per peptide) for 24 h. The plates were washed and incubated with biotinylated detection antibodies (U‐CyTech), followed by ALP‐conjugated streptavidin (U‐CyTech). The plates were developed using NBT/BCIP substrate (Pierce). Finally, plates were scanned and counted on a CellularTechnologies Limited Immunospot Analyzer.

### 4.13 Peptide ELISA

N‐terminally biotinylated SH peptide of SARS‐CoV‐2 BA.2.86 variants (BA.2.86), SARS‐CoV, OC43, HKU1, MHV, and MERS‐CoV were synthesized by GenScript. In brief, 96‐well ELISA plates were coated with 100 µL streptavidin (Biolegend, 405150‐1 mg) (2 µg/mL) at 4°C overnight. The plates were washed with PBST (0.05% Tween 20 in 1 × PBS) and blocked using 3% BAS in PBST at room temperature for 2 h. The plates were washed with PBST and incubated with biotinylated SH peptides (1 µg/mL)) at room temperature for 2 h. The biotinylated SH peptides sequences were included in Table S2. The plates were washed with PBST and incubated with monoclonal antibodies or serially diluted mouse sera at 37°C for 1 h. Then, the plates were washed and incubated with an HRP‐conjugated goat anti‐human or anti‐mouse IgG antibody (Beyotime) at room temperature for 1 h. Finally, the plates were washed and developed with 100 µL TMB substrate and terminated with 50 µL 2 M sulfuric acid. The OD values at 450 nm (OD450) were measured. IgG titers were calculated as above.

### 4.14 Pseudovirus Production

Approximately, 10 µg packaging construct psPAX2, 10 µg luciferase reporter plasmid pLenti‐CMV Puro‐Luc, and 10 µg S plasmid lacking the C‐terminal 19 aa were co‐transfected into HEK293T cells in T‐75 flask with PEI. Six hours after transfection, the culture medium were replaced with fresh medium. The supernatants were collected 48 h post‐transfection and were purified by filtration with a 0.45 µm filter.

### 4.15 Pseudovirus‐Based Neutralization Assay

The SARS‐CoV‐2 variants, SARS‐CoV and MERS‐CoV pseudoviruses neutralization assay was generated in an approach similar to that described previously [44]. To determine the neutralization activity of the serum and antibody, HEK293T‐hACE2 cells or HEK293T‐hDPP4 were seeded in 96‐well cell culture plates at a density of 2.0 × 10^4^ cells/well overnight. Note that 60 µL of three‐fold serial dilutions of heat‐inactivated samples or antibodies were prepared in 96‐well U‐bottom tissue culture plate and mixed with 60 µL of pseudovirus. The mixture was incubated at 37°C for 1 h. It was then added to HEK293T‐hACE2 or HEK293T‐hDPP4 cells. Moreover, 48 h after infection, cells were lysed in Steady‐Glo Luciferase Assay (Promega) according to the manufacturer's instructions. SARS‐CoV‐2 variants, SARS‐CoV and MERS‐CoV neutralization titers were defined as the sample dilution at which a 50% reduction in the relative light unit (RLU) was observed relative to the average of the virus control wells.

### 4.16 Cell‐Cell Fusion Assay

Vero E6 cells were transfected with S protein expression plasmids lacking the C‐terminal 19 aa, together with an equal mass of pEGFP‐C2 plasmid, using Lipofectamine 2000 according to the manufacturer's instructions. Six hours after transfection, the medium was replaced with DMEM supplemented with 5% FBS. Where indicated, antibodies CV3‐25 or CB6 were added to the culture medium at the specified concentrations. Twenty‐four hours post‐transfection, cell‐cell fusion was assessed by monitoring syncytium formation using a fluorescence microscope (Olympus CKX53), and representative images were acquired.

### 4.17 Competitive ELISA

A total of 96‐well ELISA plates were coated with 100 µL CV3‐25 antibody (1 µg/mL) at 4°C overnight. The plates were washed with PBST and blocked using 5% skimmed milk in PBST at room temperature for 1 h. Mouse sera were diluted 1:50 and JN.1 His tagged S protein (50 ng) was incubated at 4°C for 4 h. The plate was washed with PBST and incubated with the mixture of sera and protein at room temperature for 1 h. Subsequently, the plates were washed and incubated with HRP‐conjugated His‐Tag monoclonal antibody (HRP‐66005, proteintech) at room temperature for 1 h. Finally, the plates were washed and developed with 100 µL TMB substrate and terminated with 50 µL 2 M sulfuric acid. The OD values at 450 nm (OD450) were measured. IgG titers were calculated as the reciprocals of the maximal dilutions at which the OD450 values were equal to or higher than the cut‐off value (2.1 times that from the negative control wells).

### 4.18 BLI Analysis

The binding kinetics and affinities of hACE2‐Fc against JN.1 RBD and RHS were assessed by BLI (8‐channel Octet R8 system, Sartorius). All steps were performed at 30°C, 1000 rpm. Anti‐his biosensors were pre‐equilibrated in Q buffer and captured 20 µg/mL of JN.1 RBD or RHS for 120 s. After equilibrating in Q buffer for 30 s, the sensors were incubated with serially diluted hACE2‐Fc protein, or Q buffer (as control) for 300 s, and finally dissociated in Q buffer for 600 s. The association (*K*_a_), dissociation (*K*_d_), and equilibrium dissociation (*K*_D_) constants were calculated using Octet Analysis System software. Raw data and fits were plotted in GraphPad Prism 10.0.

### 4.19 SARS‐CoV‐2 Challenge Experiment

Note that 6–8 weeks female K18‐hACE2 mice were inoculated IN with 50 µl DMEM containing 10^5^ PFU SARS‐CoV‐2 Omicron JN.1 or EG.5.1 variants. At 3 days postinfection (dpi), mice were euthanized, and lung tissues were collected for hematoxylin and eosin (H&E) staining and viral load determination. The SARS‐CoV‐2 challenge experiment was conducted in BSL‐3 laboratory of Guangzhou National Laboratory.

### 4.20 Viral Load Quantification

The collected mouse lung tissue was homogenized within PBS buffer. The homogenate was added to TRIzol reagent (Invitrogen) for RNA extraction. SARS‐CoV‐2‐specific qRT‐PCR assays were performed using a One Step Probe RT‐qPCR kit (Accurate Biotech, China) on a CFX384 Touch real‐time PCR detection system (Bio‐Rad, USA) according to the manufacturer's protocol. The primers and probe was used for detecting viral genome of SARS‐CoV‐2 variant as following: N1‐F: 5′‐AAGAAATTCAACTCCAGGCAGC‐3′; N1‐R: 5′‐GCTGGTTCAATCTGTCAAGCAG‐3′; Probe: 5′‐FAM‐TCACCGCCATTGCCAGCCA‐BHQ1‐3′.

### 4.21 Cell Counting Kit‐8 (CCK‐8) Cell Viability Assay

Briefly, HEK293T cells were seeded into 96‐well plates and cultured overnight at 37°C in a 5% CO_2_ incubator. Cells were then treated with RBD or RHS proteins at final concentrations of 10 or 100 µg/mL. After incubation for 6, 12, 24, or 48 h, 10 µL of CCK‐8 solution (Vazyme, A311‐01) was added to each well and incubated for an additional 1 h at 37°C. Absorbance at 450 nm was measured using a Synergy H1 microplate reader (Agilent BioTek, Beijing, China).

### 4.22 Focus‐Forming Assay

The focus‐forming assay (FFA) was performed to quantify viral titers in lung tissues as previously described [45]. Briefly, Vero‐E6 cells were seeded into 96‐well plates and cultured overnight at 37°C in a 5% CO_2_ incubator. Lung homogenates were serially diluted and added to the cells, followed by incubation for 24 h. Cells were then fixed with 4% paraformaldehyde for 30 min and permeabilized with 0.2% Triton X‐100. After blocking with 1% bovine serum albumin (BSA) in PBS for 30 min, cells were incubated with rabbit anti‐SARS‐CoV‐2 N protein polyclonal antibody (Sino Biological, 40588‐R0002‐100) at 37°C for 1 h. Following three washes with PBST (PBS containing 0.05% Tween‐20), cells were incubated with horseradish HRP‐conjugated goat anti‐rabbit secondary antibody (Jackson, 109‐035‐088). Foci were visualized using TrueBlue peroxidase substrate (KPL) and quantified using an ELISPOT reader (Cellular Technology Ltd.).

### 4.23 SARS‐CoV‐2 and MERS‐CoV Authentic Virus Neutralization Assay

The FRNT was performed to assess serum neutralizing activity. Serially diluted sera were co‐incubated with 200 focus‐forming units (FFU) of SARS‐CoV‐2 variants or MERS‐CoV at 37°C for 1 h. The mixtures were then added to Vero E6 cell monolayers and incubated for 1 h to allow viral adsorption. Following infection, the inoculum was removed, and cells were overlaid with minimum essential medium (MEM) containing 1.6% carboxymethylcellulose (CMC) and incubated for 24 h. Cells were then fixed with 4% paraformaldehyde and permeabilized for immunostaining. Viral foci were detected using rabbit anti‐SARS‐CoV‐2 N polyclonal antibody (Sino Biological, 40588‐R0002‐100) or mouse anti‐MERS‐CoV N monoclonal antibody (Sino Biological, 40068‐MM10), followed by incubation with horseradish HRP‐conjugated goat anti‐rabbit IgG (Jackson, 111‐035‐144) or goat anti‐mouse IgG (Sino Biological, SSA007). Foci were visualized using TrueBlue peroxidase substrate (Seracare Life Sciences, 5510‐0030) and imaged using a CTL ImmunoSpot S6 Ultra analyzer (Cellular Technology Limited). Half‐maximal focus reduction neutralization titers (FRNT_50_) were calculated using GraphPad Prism.

### 4.24 Criteria for Lung Pathology Scoring

Score 0: *Normal* (intact alveolar architecture with no inflammatory cell infiltration). Score 1: *Minimal injury* (mild focal inflammatory infiltration with preserved alveolar architecture). Score 2: *Mild injury* (multifocal inflammatory infiltration with mild alveolar septal thickening and no significant consolidation). Score 3: *Moderate injury* (widespread or confluent inflammatory infiltration, marked alveolar septal thickening, and evident alveolar structural damage). Score 4: *Severe injury* (diffuse and dense inflammatory infiltration, severe destruction of alveolar architecture, extensive consolidation involving most of the field, and near‐complete loss of normal lung structure).

### 4.25 Histopathology and Immunohistochemistry

Briefly, lung tissues were fixed in 4% paraformaldehyde, embedded in paraffin, and sectioned into 4‐µm‐thick slices. Sections were subjected to H&E staining to assess tissue pathology. For immunohistochemistry (IHC), sections were deparaffinized, rehydrated, and subjected to antigen retrieval in citrate buffer. After blocking, sections were incubated with rabbit anti‐SARS‐CoV‐2 N protein antibody (Sino Biological, 40588‐R0002‐100), followed by HRP‐conjugated secondary antibody. Signals were visualized using DAB substrate and counterstained with hematoxylin. Slides were digitized using an Aperio CS2 scanner (Leica Biosystems), and images were processed using NDP.view2 software.

### 4.26 Statistical Analysis

Comparisons between groups were performed using one‐way ANOVA, two‐way ANOVA, or a one‐tailed unpaired *t*‐test. Statistical analysis was calculated using GraphPad Prism version 10. The asterisks stand for significant differences (*p < 0.05; **p < 0.01; ***p < 0.001; ****p < 0.0001). “ns” indicates no significant difference.

## Author Contributions

D.G., J.Y. and X.S. conceived the study. D.G. and J.Y. supervised the research. X.S. designed the experiments, analyzed the data, and wrote the manuscript. X.S. and J.F. performed the cell immune analysis. X.S., X.X., and Y.S. performed the plasmids construction and ELISA detection. X.S., X.L., and X.X. performed protein expression and purification. X.S. and P.H. performed BLI assay. X.S., X.X., S.Y., T.X., and K.S. performed the animal experiments and viral infections. R.C. and T.C. performed serum neutralization titer measurements in the BSL‐3 laboratory. X.M., C.L., S.L., C.X., Y.Y., and S.C. helped with reagents and discussions. D.G. and J.Y. helped with funding acquisition, and Writing – reviewing.

## Conflicts of Interest

The authors declare no conflicts of interest.

## Supporting information




**Supporting File**: advs77384‐sup‐0001‐SuppMat.docx.

## Data Availability

All data relevant to the study are available from the corresponding author upon reasonable request.
