# Supplementary material for: An Engineered HR1‐Stem Helix‐HR2 Trimeric Platform for Developing Broad‐Spectrum Coronavirus Vaccines
Source: Adv Sci (Weinh). 2026 Sep 1:e77384. Online ahead of print. doi: 10.1002/advs.77384 (PMC13534781; doi:10.1002/advs.77384)
Supplement: Supplementary file 1 [file ADVS-9999-e77384-s001.docx]

Supporting Information

**Title:**

An engineered HR1-stem helix-HR2 trimeric platform for developing broad-spectrum coronavirus vaccines

**Authors:**

*^1,2^Xikui Sun, ^2^Junhao Fan, ^1^Xiaolu Xie, ^1,2^Yuting Shi, ^2,3^Xiaoqing Liu, ^1^Tiefeng Xu,^，2^Sidi Yang, ^1^Keda Shi, ^2^Chengshu Xie, ^4^Shixiong Li, ^1^Siwen Chen, ^1,2^Ruoxi Cai, ^2^Ping He, ^2^Yuzhen Ye, ^2^Tao Chen, ^1,2^Xiancai Ma, ^5^Chunmei Li, ^1,2^Jingyou Yu*, ^1,2,3^Deyin Guo**

^1^State Key Laboratory of Respiratory Disease, National Clinical Research Center for Respiratory Disease, Guangzhou Institute of Respiratory Health, The First Affiliated Hospital of Guangzhou Medical University, Guangzhou 510182, China

^2^Guangzhou National Laboratory, Guangzhou International Bio-Island, Guangzhou 510005, China

^3^Key Laboratory of Tropical Disease Control of Ministry of Education, Institute of Human Virology, Department of Pathogen Biology and Biosecurity, Zhongshan School of Medicine, Sun Yat-Sen University, Guangzhou 510080, China

^4^College of Life Sciences, Nankai University, Tianjin 300071, China

^5^Centre for Infection and Immunity Study (CIIS), School of Medicine, Shenzhen Campus of Sun Yat-Sen University, Shenzhen 510275, China

Correspondence:

Jingyou Yu ([yu_jingyou@gzlab.ac.cn](mailto:yu_jingyou@gzlab.ac.cn)); Deyin Guo ([Guo_deyin@gzlab.ac.cn](mailto:Guo_deyin@gzlab.ac.cn))

**The supporting information includes:**

**Figure S1 to S8,**

**Tables S1-S2**

**Figure S1**. **Binding kinetics of hACE2-Fc to RBD were analyzed by bio-layer interferometry (BLI)**

Sensorgrams and corresponding kinetic fits are shown from one representative experiment. The values of association rate constant (Kon), dissociation rate constant (K_dis_), and equilibrium dissociation constant (K_D_) are shown. BLI measurements were repeated in at least two independent experiments with similar results.

**Figure S2 Determining the cell immunity by** **enzyme-linked immunospot (ELISpot) and flow cytometry**

A) Frequencies of interferon-γ (IFN-γ)-secreting (left) cells and interleukin-4 (IL-4)-secreting (right) cells in splenocytes by ELISpot. Median ± IQR are shown (n = 4 for phosphate-buffered saline (PBS) group, n=5 for other groups). B) Gating strategies for CD4⁺ and CD8⁺ T cells analysis. For CD4⁺ cells, IFN-γ, tumor necrosis factor-α (TNF-α), interleukin-2 (IL-2), and IL-4 secreting cells were analyzed. For CD8⁺ T cells, IFN-γ, TNF-α, and IL-2 secreting cells were analyzed. Statistical comparisons were performed by one‑way ANOVA with Tukey's multiple‑comparison test (A). ns, no significant difference. Data from one representative experiment are shown, and the experiment was repeated at least twice independently with similar results.

**Figure S3 The neutralization activity of CV3-25 against sarbecoviruses**

The neutralizing activity of CV3-25 was assessed using pseudotyped viruses representing SARS-CoV-2 D614G variant, SARS-CoV-2 Omicron subvariants BA.5, XBB.1.5, EG.5.1, BA.2.86, JN.1, SLip, KP.2, KP.3, KP.2.3, LB.1, XEC, and KP.3.1.1, SARS-CoV, and WIV1. The half-maximal inhibitory concentration (IC_50_) values were fitted using GraphPad Prism and were shown. Data from one representative experiment are shown. The assay was repeated in at least two independent experiments with similar results.

**Figure S4 Inhibitory activity of CV3-25 against SARS-CoV-2 spike (S)-mediated membrane fusion**

The inhibitory activity of CV3-25 was evaluated using a cell-cell fusion assay. Vero E6 cells were co-transfected with equal amounts of S protein expression plasmids lacking the C-terminal 19 aa and pEGFP-C2 using Lipofectamine 2000. Six hours post-transfection, the medium was replaced with Dulbecco’s modified Eagle medium (DMEM) supplemented with 5 % fetal bovine serum. CB6 (final concentration 150 μg/mL) or CV3-25 (final concentration 4 μg/mL) antibodies were added to the cultures to assess inhibition of membrane fusion. Cell-cell fusion was visualized and imaged by fluorescence microscopy 24 h after transfection. Data from one representative experiment are shown. The data were repeated in three independent experiments with similar results.

**Figure S5 Characterization of RHSΔ6-aa and RHSΔSH trimer protein**

A) Western blot analysis of JN.1 RBD, RHS, RHSΔ6-aa, or RHSΔSH expression in the culture supernatants of HEK293T cells 48 h post-transfection in non-denaturing and denaturing conditions. For denaturing conditions, supernatants were mixed with 5 × sodium dodecyl sulfate-polyacrylamide gel electrophoresis (SDS-PAGE) protein loading buffer containing β-mercaptoethanol and heated at 100 °C for 10 min prior to SDS-PAGE analysis. For non-denaturing conditions, supernatants were mixed with 5 × SDS-PAGE protein loading buffer without β-mercaptoethanol and analyzed without heat treatment. B) size-exclusion chromatography (SEC) profiles of RHSΔ6-aa (left) and RHSΔSH (right) using a HiLoad 16/600 Superdex 200 pg or Superdex 200 Increase 10/300 GL column under native, non-denaturing conditions. The UV 280 nm absorbance curves are indicated. C) Ultracentrifugation sedimentation profiles of RHSΔ6-aa (left) and RHSΔSH (right). D) The structure of RHSΔ6-aa (left) and RHSΔSH (right) predicted by AlphaFold3. E) The binding activity of RHS, RHSΔ6-aa, or RHSΔSH to hACE2-Fc at indicated concentration determined by enzyme-linked immunosorbent assay (ELISA). Data points represent mean ± SD (n = 3) from three independent experiments. Statistical comparisons were performed by two‑way ANOVA (E). ns, no significant difference. Western blot, SEC, and analytical ultracentrifugation data were repeated in at least two independent experiments with similar results. The data are shown from one representative experiment.

**Figure S6 SEC profiles of RHS-1, RHS-2, S-2P, and RBD-HR**

A) SEC profiles of RHS-1, RHS-2, S-2P, and RBD-HR were analyzed using a HiLoad 16/600 Superdex 200 pg or Superdex 200 Increase 10/300 GL column under native, non-denaturing conditions. The UV 280 nm absorbance curves are indicated. The SDS-PAGE analysis of the purified RHS-1, RHS-2, S-2P, or RBD-HR protein in non-denaturing and denaturing conditions were shown on the right of each corresponding SEC. For denaturing conditions, samples were mixed with 5 × SDS-PAGE protein loading buffer containing β-mercaptoethanol and heated at 100 °C for 10 min prior to SDS-PAGE analysis. For non-denaturing conditions, samples were mixed with 5 × SDS-PAGE protein loading buffer without β-mercaptoethanol and analyzed without heat treatment. B) Representative transmission electron microscopy (TEM) micrographs of RHS, RHS-1, and RHS-2 (top) and the corresponding 2D classification analysis (bottom). The data were repeated in at least two independent experiments with similar results. And the data are shown from one representative experiment.

**Figure S7 Safety evaluation of the RHS vaccine.**

A) Cell viability of HEK293T cells treated with RBD or RHS proteins. Cells were incubated with 10 or 100 μg/mL of RBD or RHS for 6, 12, 24, and 48 h, and viability was assessed using a Cell Counting Kit-8 (CCK-8) assay. Data are presented as mean ± SD (n = 3) from one representative experiment. The experiment was repeated twice with similar results. B) Body weight changes of BALB/c mice immunized intramuscularly (IM) with 10 μg of RBD or RHS formulated with AddaVax adjuvant over 6 days. Control mice received an equal volume of PBS. Data are presented as mean ± SD (n = 5). C-E) Representative hematoxylin and eosin (H&E) staining of heart (C), liver (D), and kidney (E) sections from mice 48 h post-immunization with 10 μg of RBD or RHS formulated with AddaVax. F) Serum levels of alanine aminotransferase (ALT), aspartate aminotransferase (AST), alkaline phosphatase (ALP), urea, and creatinine (CREA) measured at 0.25, 1, and 6 days post-immunization. Data are presented as mean ± SD (n = 5). The in vivo safety evaluation was conducted in a single independent experiment with five mice per group. Statistical comparisons were performed by one‑way ANOVA (A, B, and F) with Tukey's multiple-comparison test. ns, no significant difference.

**Figure S8 Characterization of SARS-CoV and MERS-CoV RBD and RHS protein expression**

A) Western blot analysis of RBD and RHS expression of SARS-CoV (left) and MERS-CoV (right) in the culture supernatants of HEK293T cells 48 h post-transfection in non-denaturing and denaturing conditions. B) SEC profiles of SARS-CoV RBD under native, non-denaturing conditions. The UV 280 nm absorbance curves are indicated. The SDS-PAGE analysis of the purified SARS-CoV monomer and dimer RBD protein in non-denaturing and denaturing conditions were shown on the right of SEC. C) The SDS-PAGE analysis of the purified MERS-CoV RBD and RHS protein in non-denaturing and denaturing conditions. D) The structure of SARS-CoV RHS (left) and MERS-CoV RHS (right) predicted by AlphaFold3. E) SEC profiles of OVA-JN.1-HS, ovalbumin (OVA)-SARS-CoV-HS, and OVA-MERS-CoV-HS using Superdex 200 Increase 10/300 GL column under native, non-denaturing conditions. Dashed lines indicate the SEC fractions collected for subsequent SDS-PAGE analysis. F) SDS-PAGE analysis of SEC fractions of OVA-JN.1-HS, OVA-SARS-CoV-HS, and OVA-MERS-CoV-HS in non-denaturing and denaturing conditions. Fractions are arranged from left to right according to their order of collection. For denaturing conditions in Western blot and SDS-PAGE analysis, samples were mixed with 5 × SDS-PAGE protein loading buffer containing β-mercaptoethanol and heated at 100 °C for 10 min prior to SDS-PAGE analysis. For non-denaturing conditions in Western blot and SDS-PAGE analysis, supernatants were mixed with 5 × SDS-PAGE protein loading buffer without β-mercaptoethanol and analyzed without heat treatment. Western blot, SEC, and SDS-PAGE data were repeated in at least two independent experiments with similar results. The data are shown from one representative experiment.

**Table S1 Solvent-accessible surface area (SASA) analysis of RBD exposure in RHS-derived protein**

| Name of Protein | Type of linker | RBD exposure | RBD exposure  Change (%) | Spatial hindrance | Spatial hindrance  change (%) |
| --- | --- | --- | --- | --- | --- |
| RHS | flexible | 0.95 | 0 | 0.13 | 0 |
| RHS-1 | no | 0.55 | -42.3 | 0.2 | 57 |
| RHS-2 | rigid | 0.72 | -23.5 | 0.18 | 41.8 |

**Table S2 The sequence of the biotinylated β-coronavirus SH**

| β-coronavirus | SH sequences |
| --- | --- |
| MERS-CoV | Biotin-PLLGNSTGIDFQDELDEFFKNVSTSIP |
| HKU1 | Biotin-HSVPKLSDFESELSHWFKNQTSIAP |
| OC43 | Biotin-TSIPNLPDFKEELDQWFKNQTSVAP |
| MHV | Biotin-NSIPNLPDFKEELDQWFKNQTSIAP |
| SARS-CoV | Biotin-PLQPELDSFKEELDKYFKNHTSPDV |
| SARS-CoV-2 JN.1 | Biotin-PLQLELDSFKEELDKYFKNHTSPDV |
